# Fouling-Resistant Voltammetric Xylazine Sensors for Detection of the Street Drug “Tranq”

**DOI:** 10.3390/toxics12110791

**Published:** 2024-10-30

**Authors:** Joyce E. Stern, Ann H. Wemple, Charles W. Sheppard, Arielle Vinnikov, Michael C. Leopold

**Affiliations:** Department of Chemistry, Gottwald Center for the Sciences, University of Richmond, Richmond, VA 23173, USAcharlie.sheppard@richmond.edu (C.W.S.); arielle.vinnikov@richmond.edu (A.V.)

**Keywords:** xylazine, fentanyl, opioid, adulterant, Tranq, Zombie, differential pulse voltammetry

## Abstract

As the opioid crisis continues to wreak havoc on a global scale, it is increasingly critical to develop methodologies to detect the most dangerous drugs such as fentanyl and its derivatives, which have orders of magnitude higher potency than morphine. The scientific challenge for chemical detection of fentanyl and its derivatives is complicated by both the constantly increasing synthetic variations of the drug as well as the expanded use of adulterants. One tragically consequential example is the nocuous street drug known as “Tranq”, which combines fentanyl or a fentanyl derivative with the veterinary sedative Rompun^®^, chemically identified as xylazine (XYL). This pervasive street cocktail is exacerbating the already staggering number of fentanyl-related deaths as its acute toxicity poses a danger to medical first-responders and complicates their initial assessment and treatment options for overdose victims. Given the widespread use of XYL as an adulterant, an electrochemical XYL sensor capable of on-site operation by non-experts as a fast-screening tool is a notable goal. This work presents a voltammetry-based sensor featuring carbon electrodes modified with carboxylic-acid functionalized multi-walled carbon nanotubes layered with cyclodextrin and polyurethane membranes for sensitivity and selectivity enhancements. The sensor has critical and robust fouling resistance while providing sensitivity at 950 μA/mM∙cm^2^, a low limit of detection (~5 ppm), and the ability to detect XYL in the presence of fentanyl and/or other non-fentanyl stimulants like cocaine. The demonstrated sensor can be applied to promote public health with its ability to detect and indicate XYL in the presence of opioids, serving to protect drug-users, first responders, medical examiners, and on-site forensic investigators from exposure to these dangerous mixtures.

## 1. Introduction/Background

**Figure 1:**
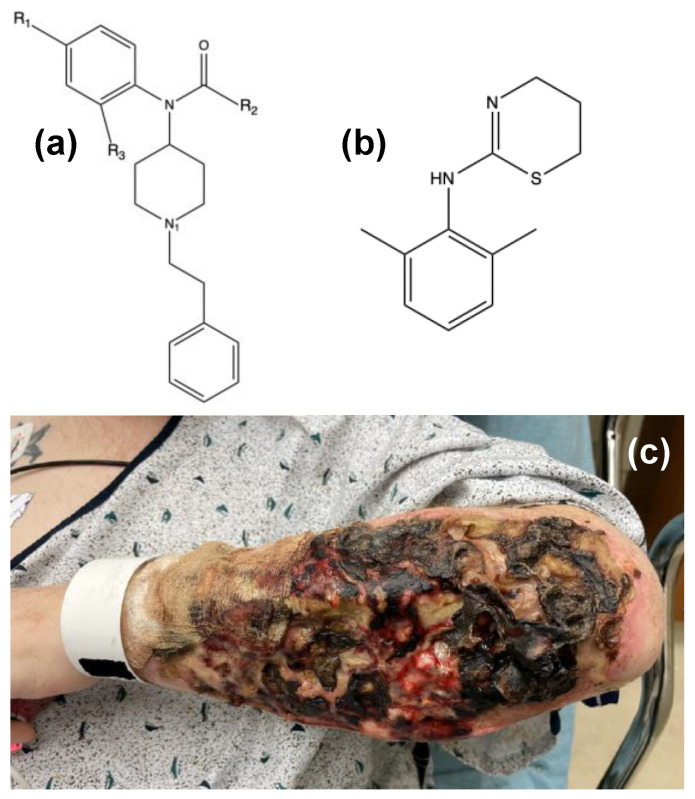
The core structure of (**a**) fentanyl where structural changes at R_1_, R_2_, and R_3_ create synthetic fentanyl analogs; (**b**) the structure of xylazine (XYL); and (**c**) the skin necrosis that can occur when misusing the street drug “Tranq,” fentanyl adulterated with XYL (from Ref. [19]).

The world-wide opioid crisis caused by growing access to narcotics presents a devasting societal burden, with high fatality rates and serious chronic medical issues that deliver catastrophic economic costs. The COVID-19 pandemic exacerbated the already disastrous effects of opioids, resulting in large increases in use, abuse, and overdoses [1,2]. In addition to the massive rise of opioid-related deaths and the accompanying interpersonal family devastation that follows, the effects of the opioid epidemic also include victims having to manage withdrawal symptoms, lack of health-care coverage leading to spiraling debt, and corresponding implications of those factors on workforce numbers and availability [3]. In recent years, the drug fentanyl (Figure 1a) has dominated the illegal opiate market, resulting in easy access to highly dangerous, unregulated substances.

Fentanyl is often attributed to an increase in fatality rates of the overall opioid epidemic [3,4] as it has a fast-acting mechanism and is more potent than other opioid options such as morphine and heroin, resulting in an exponentially growing mortality rate [5]. Furthermore, derivatives of the parent compound fentanyl can be easily synthesized (i.e., synthetic manipulation/functionalization of R1, R2, and R3 in Figure 1a) [6] to act faster or utilize different administration routes. In turn, the derivatization requires researchers to develop new treatments and detection methods as an essential provision of any prevention and mitigation strategies [6]. Challenges arise, however, in the field due to the accessibility and adaptability of cost-effective sensors, such as in medical settings and by first responders on overdose calls. While chromatography techniques have been utilized to detect fentanyl, its limitations include the high price of operation and the need for trained personnel as well as the typically non-portable nature of the instrument [7]. Electrochemical techniques, while less investigated in the literature, present an appealing alternative to addressing the limitations of other techniques [8].

Further compounding the opioid crisis is the adulteration of fentanyl and its derivatives with xylazine (Figure 1b), a chemical tranquilizer not approved for use in humans. This extremely dangerous cocktail is known as the street drug “Tranq” or “Zombie”. A synthetic non-opioid, xylazine (XYL) is typically used by veterinarians as a sedative, muscle relaxant, or for its analgesic effects. Drug dealers “cut” their fentanyl drugs with the significantly cheaper and largely accessible XYL while still maintaining the desired “high” for chronic opioid users who are often unaware of the combination of drugs [9,10,11,12,13,14,15,16]. Because XYL is intended as an intravenous drug, addicts missing their veins during self-administered “Tranq” injections suffer severe necrotic ulcers that are difficult to heal (Figure 1c) [17,18,19]. Early studies suggest that “Tranq” prolongs the XYL effect while augmenting the effects of fentanyl, providing a more desirable high that delays the negative withdrawal effects typical of other opioid drugs [12,13]. However, unlike fentanyl, XYL does not respond to naloxone as it is a nonopioid synthetic drug, presenting additional complications for first responders treating potential overdose patients [9]. While XYL has been part of “street drugs” additives for decades, recent studies illuminate a stark increase in overdose deaths involving XYL. Most notably from the studies, in almost all cases (98%), XYL was present with fentanyl or a fentanyl derivative [12,14,20]. As such, the development of an effective XYL sensor would have the secondary benefit of also quickly indicating a high probability of the presence of fentanyl or fentanyl derivatives [20]. It should be noted that XYL has also been implicated in sexual assaults as a “date rape” drug. Used alone or in combination with an opioid, XYL doping can cause muscle weakness and prolonged blackouts that make a person susceptible to sexual assault [21,22].

While traditional laboratory-based techniques for qualitative analysis of dangerous opioids remain effective, with new methodologies under constant development, the toxicity of these chemicals pose a risk to first-responders and forensic crime scene investigators, suggesting that it would be beneficial to develop point-of-use, preliminary or presumptive screening tests for the fast, on-site identification of dangerous substances [23,24,25]. The analytical methods that lend themselves to testing that is fast, inexpensive to mass produce, and usable by non-experts often involve electrochemical and/or colorimetric sensing schemes that can be miniaturized for on-site usage [8,26]. In many of these cases, a common strategy employed by researchers is to incorporate various nanomaterials (NMs) into sensing schemes for the purpose of signal enhancement [27,28,29].

Overshadowed by research focus on fentanyl and its many synthetic analogs (i.e., fentalogues), the electroactive drug XYL being present in most street fentanyl-based drugs like “Tranq” or “Zombie” represents an opportunity that has received significantly less attention in the literature until recently. In 2019, Mendes et al. published seminal work on XYL electrochemistry that produced several important findings [30]. The study showed the most sensitive electrochemical activity for XYL at clean glassy carbon electrodes (GCE), though they eventually became significantly fouled during voltametric scanning. The study successfully demonstrated the use of differential pulse voltammetry (DPV) for quantifying XYL in pharmaceuticals and urine but required the unfortunate step of polishing the GCEs prior to every scan, thereby limiting its on-site application. As with most sensing targets, researchers have also employed various NMs within XYL sensing schemes. Notable examples of this approach include the work of El-Shal using cyclic voltammetry (CV) at electrodes modified with an ionic liquid composite film containing multi-walled carbon nanotubes (MWCNTs) [31]. Saisahas et al. published two papers on portable electrochemical XYL sensors [32,33]. First, graphene nanoplatelets on screen printed carbon electrodes were used with DPV to detect XYL in beverages with precent recoveries ranging from 80 to 108% [33]. The second report performed DPV with nanocoral-modified graphene paper electrodes for XYL detection with similar precent recoveries [32]. Both reports showed calibration curves with two linear ranges having a higher sensitivity at low XYL concentrations and a more depressed sensitivity at higher XYL concentrations, trends again attributed to the inherent electrode fouling during XYL electrolysis. Interestingly, while these reports seem to focus on XYL oxidation at the electrode, there is no consensus on the exact electrochemical mechanism [30,31,32,33]. One of the more common mechanisms found in the literature is shown in the Supplementary Data (Scheme S1). Notably, these reports all focus on the direct detection of XYL in samples rather than its detection as a street drug adulterant (i.e., in presence of fentanyl).

This study presents a versatile and fouling-resistant sensing scheme for electrochemical detection of XYL that meets the criteria for an effective, point-of-use, preliminary screening method for the identified applications. The scheme builds off prior work from our group that demonstrated that film-modified electrodes showed significantly enhanced sensitivity with the incorporation of MWCNTs and improved selectivity from harnessing host-guest chemical interactions using cyclodextrins in conjunction with semi-permeable membranes [34,35]. Most importantly, these fouling-resistant sensors are demonstrated to be effective in XYL detection in the presence of fentanyl and other opioids as an adulterant, making it a promising tool to protect first responders, innocent bystanders, and addicts by quickly identifying these increasingly prevalent and highly dangerous street drug mixtures.

## 2. Experimental Details

### 2.1. Materials and Instrumentation

Chemicals were purchased from reputable chemical vendors in high purity and used as received whenever possible. Chemical solutions were all made using ultra-purified water (18.2 MΩ∙cm). Polyurethanes of hydrothane (HPU, AL25-80A) and Tecoflex (TPU, SC-80A) were obtained from AdvanSource Biomaterials (Wilmington, MA, USA) and Lubrizol (Cleveland, OH, USA), respectively. Carboxylic-acid derivatized multi-walled carbon nanotubes (COOH-MWCNT) and β-cyclodextrin (β-CD) molecules were purchased from Nano Lab Inc. (Waltham, MA, USA) and Ambeed, Inc. (Arlington Heights, IL, USA). A Branson sonicator (Model 2510; 40 kHz; 130W) was used for pretreatment of the COOH-MWCNTs. For electrochemical experiments, glassy carbon electrodes (GCE), Ag/AgCl reference electrodes from CH Instruments (Bee Cave, TX, USA) with platinum coiled wire counter electrodes (Millipore-Sigma, St. Louis, MO, USA) were used with 8-channel model 1000B or 1030C potentiates from CH Instruments. Xylazine (XYL) was obtained from Chem-Impex International (Wood Dale, IL, USA) through VWR International, LLC and freshly prepared prior to use (50 mM standard solutions). Fentanyl and cocaine were both purchased from Cerilliant (Round Rock, TX, USA). Popular name-brand beverages were purchased locally at supermarkets and Virginia ABC stores with potential interferent chemicals ordered through traditional vendors: aspartame, phenylalanine, Acesulfame, caffeine, citric acid, sucrose, and glucose (Millipore-Sigma/Supleco).

### 2.2. Sensor Fabrication

The general procedure for sensor fabrication mimicked that of prior work in the lab [35]. Briefly, GCEs that had been polished using successively smaller alumina powder (1.0, 0.3, and 0.05 µm) in ultra-pure water suspensions on cloth plates (Buehler, Lake Bluff, IL, USA) affixed to a polishing wheel were subsequently rinsed thoroughly and dried with a N_2_ gas stream prior to modification. In preparation for sensor fabrication, two solution mixtures were prepared: a mixture of COOH-MWCNTs (2 mg) and β-CD (2 mg) was created in 1 mL of ethanol (200 proof) and sonicated (30 min) and a polyurethane (PU) blended solution comprised of HPU (75 mg) and TPU (25 mg) in ethanol:THF (1:1, 5 mL) which was stirred vigorously overnight. For the optimized sensor composition, freshy polished GCE electrodes were modified via micropipette depositions of COOH-MWCNT with β-CD (7 µL) followed by the PU blend solution (10 µL) with a drying time of 10 min for each layering. Modified electrodes were soaked in 150 mM potassium phosphate buffer (PBS, pH 7) solution (15 min) prior to being transferred to fresh PBS (25 mL) for electrochemical testing. Notably, prior to incorporating it into the sensing scheme, the host-guest binding chemistry of XYL with β-CD was confirmed by comparing differential pulse voltammetry (DPV) scans of these modified electrodes in solutions of XYL with the presence and absence of di(2-ethylhexyl) phthalate (DEHP), a known strong binder to β-CD cavities (Supplementary Data, Figure S1) [36,37].

### 2.3. Sensor Testing and Preparation—Beverages and Opioids

DPV was the primary electrochemical technique applied to the modified electrodes, with the following standard parameters employed in select potential windows: potential increment (0.004 V), amplitude (0.07 V), pulse width (0.05 s), sample width (0.0167 s), and a pulse period (0.5 s). For calibration curves, XYL injections were followed by 3 min of stirring and at least 3 min of quiet time (i.e., quiescent solution) prior to DPV measurements. For the analysis of beverages, test solutions were prepared by first opening the product and, if necessary (e.g., sodas) removing carbonation with agitation or leaving the bottle uncapped overnight. Because XYL oxidation is known to be pH dependent, addition of 2.5 M NaOH dropwise was used to neutralize the soda samples to pH ~7. Alcoholic beverages were diluted 1:1 with the 150 mM PBS to simulate a mixed cocktail (e.g., 12.5 mL each). Fentanyl and cocaine laced solutions were prepared from solids obtained from dried contents of ampules of methanol and acetonitrile solutions (1 mg/mL), respectively.

## 3. Results and Discussion

**Figure 2:**
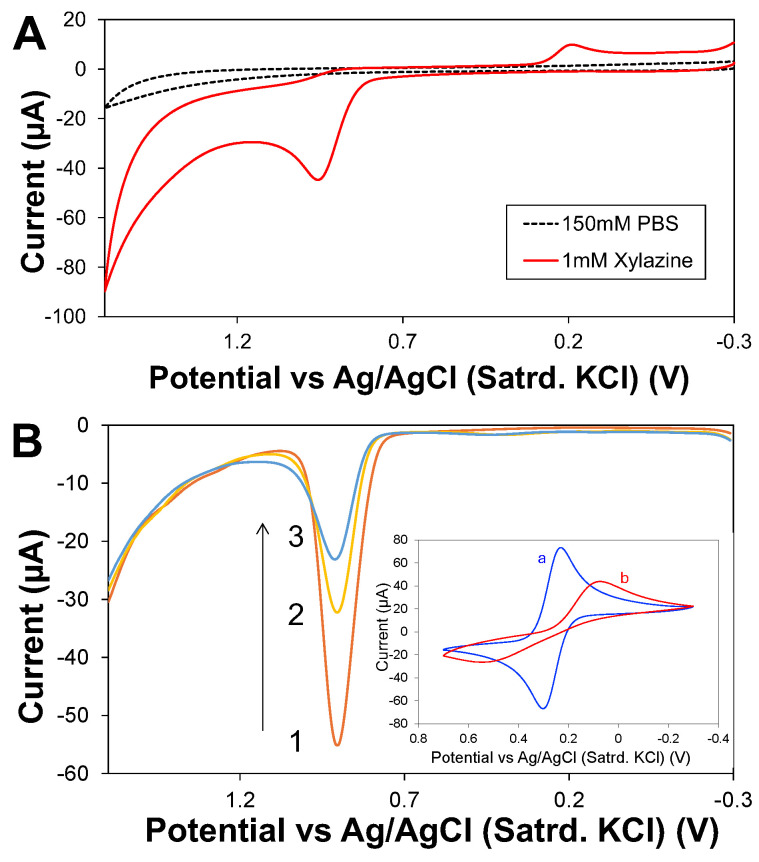
(**A**) Cyclic voltammetry (CV) of 1 mM XYL (*solid trace*) and corresponding background (*dashed trace*) of PBS without XYL (150 mM at pH = 7; 100 mV/s); (**B**) first three consecutive DPV oxidation scans of bare GCE in 1 mM XYL in PBS (150 mM PBS at pH = 7); Inset: CV of 5 mM potassium ferricyanide in 0.5 KCl at unmodified GCE (a) before and (b) after XYL exposure under applied oxidative potentials (100 mV/s).

The design and development of any voltammetry-based sensor typically begins with establishing the electrochemical behavior of the targeted analyte at an electrode interface. Prior work in the literature showed that XYL electrochemistry was most readily observed at GCEs versus traditional metallic electrodes like gold and platinum [30]. Figure 2A shows typical cyclic voltammetry (CV) for XYL at a bare GCE with irreversible peaks representing XYL oxidation at +1.0 V and the reduction of that oxidation product at +0.2 V, both of which are consistent with the literature [30]. Focusing on the oxidation of XYL, and as seen in Figure 2B, a DPV sweep toward positive potentials highlights the oxidation peak. Repeated sweeps, however, show several changes to the peak, including diminished peak current, peak broadening, and a slight shift of the peak potential—all of which suggest electrode interface fouling. CV of ferricyanide at a GCE before and after exposure to XYL oxidation scans confirms a passivated electrode consistent with fouling from XYL exposure (Figure 2B, inset). If electrochemistry is to be used for a XYL sensor, the inherent fouling during XYL oxidation presents a number of challenges, including complications during calibration, application to high concentrations, and/or re-use of the sensors. As previously mentioned, some studies circumvent this problem by cleaning/polishing the electrode prior to every XYL exposure [30], an impractical and time intensive approach not conducive for point-of-use operation by non-experts.

### 3.1. XYL Sensor Fabrication via Electrode Modification

In designing a voltammetry-based sensor for XYL, sensitivity and selectivity were partially addressed using established materials, findings, and methodologies in the literature, including from our own work [35,38]. In this regard, achieving electrochemical sensitivity toward XYL was first addressed in the sensor design by incorporating multi-walled carbon nanotubes (MWCNT) for signal enhancement as well as applying a pulsed voltammetry technique. Prior work in our lab and other labs has shown the signal enhancement utility of incorporating pre-sonicated MWCNT into sensing schemes involving direct redox activity of a targeted analyte [31,34,35]. Notably, electrochemical signal enhancement via the use of NMs within modified electrodes is often accompanied by an increase in capacitive or charging current (i.e., noise) [39,40]. DPV and its inherent ability to discriminate against charging current, thereby improving signal-to-noise, has been shown to be an effective voltammetry technique for modified electrodes of this nature [31,32,41]. As such, for the current study, DPV is the primary electrochemical technique employed.

**Scheme 1:**
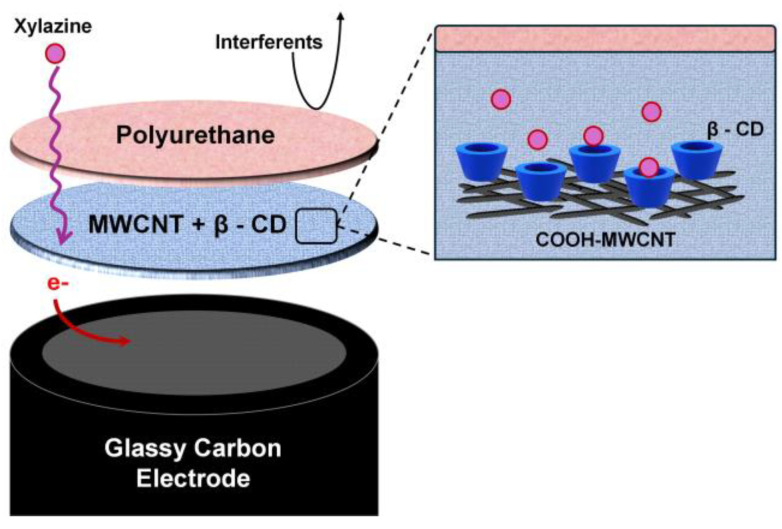
Depiction of layer-by-layer modification of GCE for XLY detection using COOH-MWCNT for sensitivity as well as β-CD molecules for host-guest chemistry and a polyurethane (PU) layer for selectivity.

Selectivity considerations for XYL were also partially addressed via previously established methodology and materials. Prior reports have shown the ability of cyclodextrins (CDs) to engage in host-guest chemical interactions for improved selectivity in sensing schemes [34,42]. A critical requirement of using CDs as host molecules in a sensor is the ability of targeted guest molecules to significantly interact as a guest within the CD cavity [43]. NMR and computational studies in the literature suggest such an interaction exists between XYL and β-cyclodextrin (β-CD) molecules [44,45]. Additionally, there has been substantial success using a blended polyurethane (PU) outer-layer for additional selectivity control in many sensor designs [46,47]. Using all these studies as the basis for an effective XYL sensor, Scheme 1 was developed and executed as described in the Experimental Details (Section 2), including the incorporation of carboxylic acid functionalized MWCNTs (COOH-MWCNTs) as a signal-enhancing NM. As detailed therein, certain parameters in the fabrication process adhered to prior findings and were not expressly optimized for the current study, including sonication times, the specific PU blend, and the β-CD-to-MWCNT ratio [34,35]. For the purposes of this study, a “layered notation” is used to describe the different compositions of electrode modification. For example, the notation GCE/COOH-MWCNT+β-CD/PU indicates a carbon electrode with sequential deposition layering of a sonicated sample mixture of COOH-MWCNT and β-CD, followed by a PU blend capping layer.

**Figure 3:**
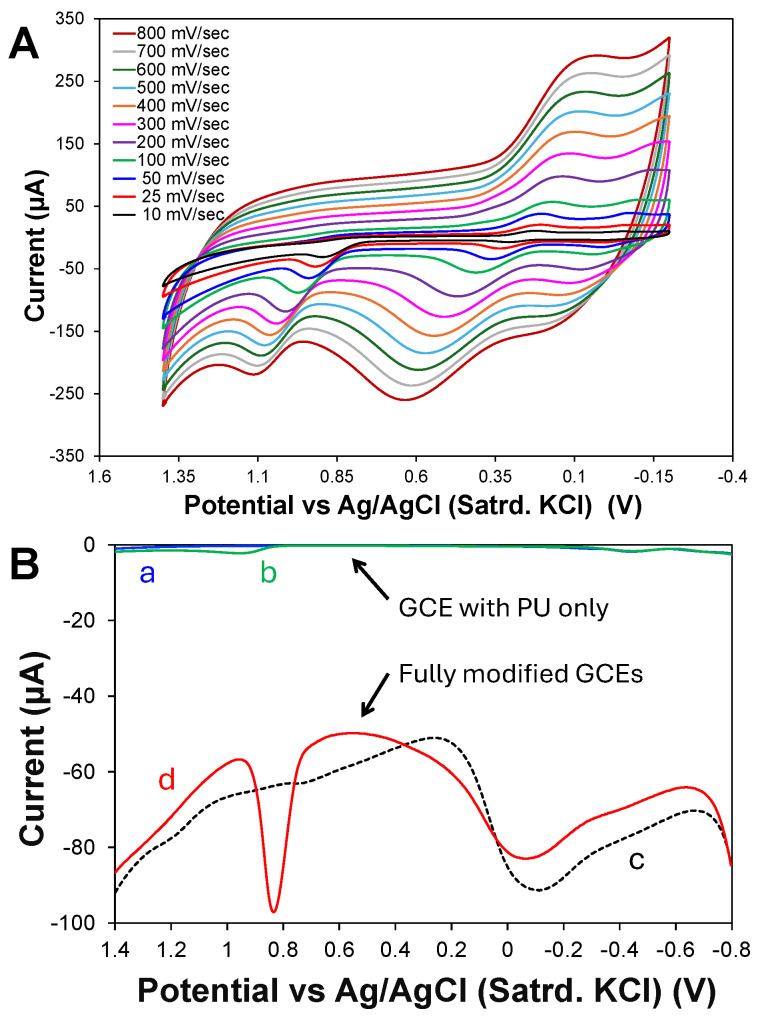
(**A**) CV of 2.5 mM XYL in PBS (150 mM; pH = 7) collected at various scan rates (inset) at the modified electrode system showing three major peaks including the primary XYL oxidation peak at +0.9 V, subsequent reduction of that product (+0.25V), and a second oxidation peak (+0.35) attributed to the MWCNTs; (**B**) DPV oxidative scans at GCEs modified with PU only (*top scans*) and fully modified GCEs (*bottom scans*) in PBS without XYL (a,c) vs. in 1 mM XYL solutions (b,d).

Initial characterization of the layers used to modify the electrode was executed using CV to systematically observe how different layers affected the XYL oxidation signal. Additionally, experiments aimed at optimizing or understanding individual contributions of different layers utilized relatively high concentrations of XYL (1 mM). CV scans of the full system (GCE/COOH-MWCNT+β-CD/PU) as well as individual components/layers like the PU, COOH-MWCNTs, and β-CD were conducted over a wide potential window. The net findings from these CV experiments, shown in Supplemental Data (Figures S2–S4), are that (a) COOH-MWCNTs significantly enhance signal, (b) certain voltametric responses are XYL concentration dependent including XYL oxidation, and (c) other peaks can be attributed to the COOH-MWCNTs (i.e., not affected by XYL concentration). Additionally, the CV shows that incorporating the COOH-MWCNTs does induce an electrocatalytic effect, shifting the XYL oxidation potential more negative compared to systems without the NMs. Given this information, a smaller potential window for CV, focused on a limited number of peaks produced using the fully modified electrode and that includes the initial XYL oxidation (Figure 3A), was analyzed for scan rate dependence as in other literature reports of XYL electrochemistry [30,31,33]. Results of the study show the XYL oxidation peak at approximately +0.95 V and subsequent XYL-related reduction peak at +0.25 V as well as the anodic peak at +0.35 V, attributed to the COOH-MWCNTs, were analyzed (potentials estimated for 10 mV/s scan, Figure 3A). The scan rate dependence, shown in Supplementary Data (Figure S5A,B and Table S1), suggests the initial oxidation of XYL is more of a diffusion-controlled process, while the subsequent reduction at +0.25 V is more of a mixed process of both diffusional and adsorbed behavior. The anodic peak at +0.35V is only present with films containing COOH-MWCNT and does not change when XYL concentration is altered. The inconclusive nature of this analysis (i.e., the mixed diffusional/adsorbed tendencies observed from the data) is not surprising and has been noted in previous reports, including calibration curves with two different linear ranges [32,33]. Both those reports and the scan rate dependence results in this study are consistent with electrochemistry at an increasingly fouled electrode. That is, the initial XYL oxidation is mostly diffusional while the subsequent reduction of that oxidized species has some adsorbed characteristics attributed to the species no longer being able to freely diffuse from the electrode because of fouling passivation. While it is understood that the β-CD interaction with XYL is likely transient [34,35,36,37], it is also recognized that XYL fouling is present and that the two processes cannot be completely separated. As such, the rest of the study was conducted under the assumption that electrode fouling was a factor and minimizing its effect on sensor function.

Figure 3B shows the DPV oxidative scans of XYL at a fully modified electrode that reiterate the CV findings where the oxidative peak at +0.85 V is attributed to XYL oxidation and is both concentration dependent and present with or without the MWCNTs, while the earlier oxidative peak at approximately +0.0 V is present in the absence of XYL. Notably, the fully modified electrode featuring MWCNTs exhibits expected increases in charging current but also a significant XYL oxidation signal even with the presence of the PU capping layer (Figure 3B, *bottom scans*). Comparatively, the XYL oxidation signal is drastically smaller (Figure 3B, *top scans*), with only a PU-modified GCE. Given this signal enhancement from MWCNTs and coupled with the semi-permeable selectivity of the PU layer, the fully modified electrode sensor design (Scheme 1) seeks to target XYL oxidation while minimizing the subsequent electrode fouling.

### 3.2. Modified Electrodes vs. Bare Electrodes—Fouling Resistance

**Figure 4:**
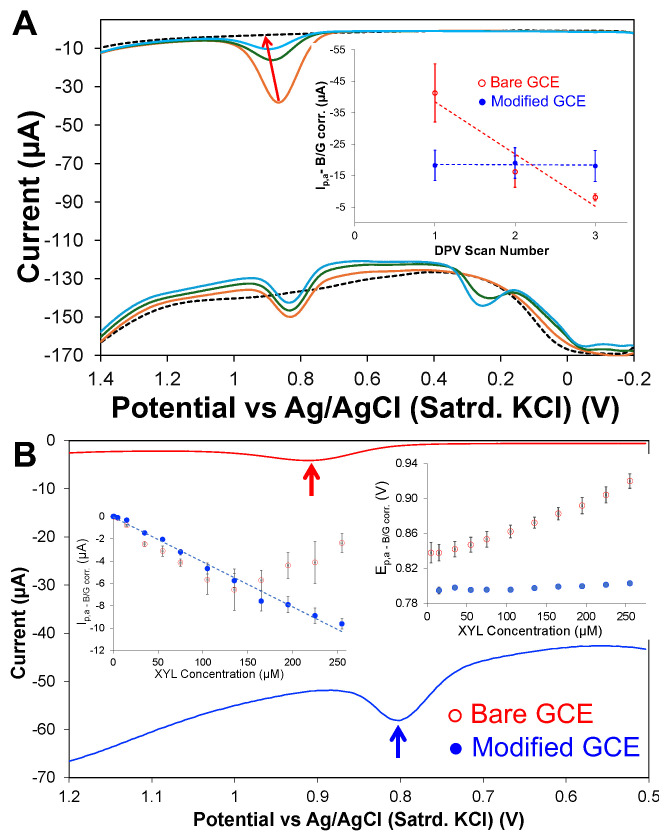
(**A**) Consecutive DPV scans of XYL (1 mM) oxidation versus background scan (*dashed*) in PBS (150 mM; pH = 7) at a bare GCE (*top scans*) showing significant decrease in peak current (*black*) and shift in peak potential (*red*) vs. successive scans at a modified GCE (*bottom scans*) and comparison of each system’s peak current changes with repeated scans (inset); (**B**) DPV of bare GCE (*top, red*) versus modified GCE (*bottom, blue*) after exposure to increasing XYL concentrations (255 μM) and comparisons of peak current (I_p,a_) and peak potential (E_p,a_) after XYL exposure (insets). Note: In some cases, error bars are smaller than markers denoting average.

As previously mentioned, the requirement of frequent cleaning/polishing of an electrode can limit the overall utility of a sensing scheme [30]. When DPV scans are repeated at a bare electrode versus the modified electrode developed in this study, the fouling-resistant nature of the system is evident. As seen in Figure 4, at the bare GCE, peak current of XYL oxidation is drastically attenuated with each scan as the fouling passivates the electrode. While not as obvious during these initial scans, the peak potential (E_p,a_) of XYL at the bare electrode is also observed to shift toward more positive potentials. Alternatively, the modified electrode maintains a well-defined peak, minimally diminished current (Figure 4A, inset), and a significantly more stable E_p,a_ from XYL oxidation over the same timeframe. The strength of the fouling-resistance of the modified electrode is most significant when DPV results over longer times and at higher XYL concentrations. Figure 4B displays DPV of XYL at both a bare GCE and the modified electrode after exposure to numerous scans at increasing XYL concentration. In this comparison, one can discern the difference in performance as the XYL peak has undergone a significant potential shift and peak broadening with diminished size/current. Tracking both the anodic peak current (I_p,a_) and E_p,a_ for both electrode systems during XYL exposure (Figure 4B, insets) shows the modified electrode is able to maintain a linear relationship with current as a function of XYL concentration and minimal shift in E_p,a_. In contrast, the bare GCE exhibits a shift in potential at low XYL concentration exposure and, while the current is linear with concentration at low concentrations, it eventually begins to diminish after exposure to ~120 µM XYL. Additional examples of this fouling-resistant behavior are provided in the Supplemental Data (Figure S6).

### 3.3. Analytical Performance of Xylazine Sensor

**Figure 5:**
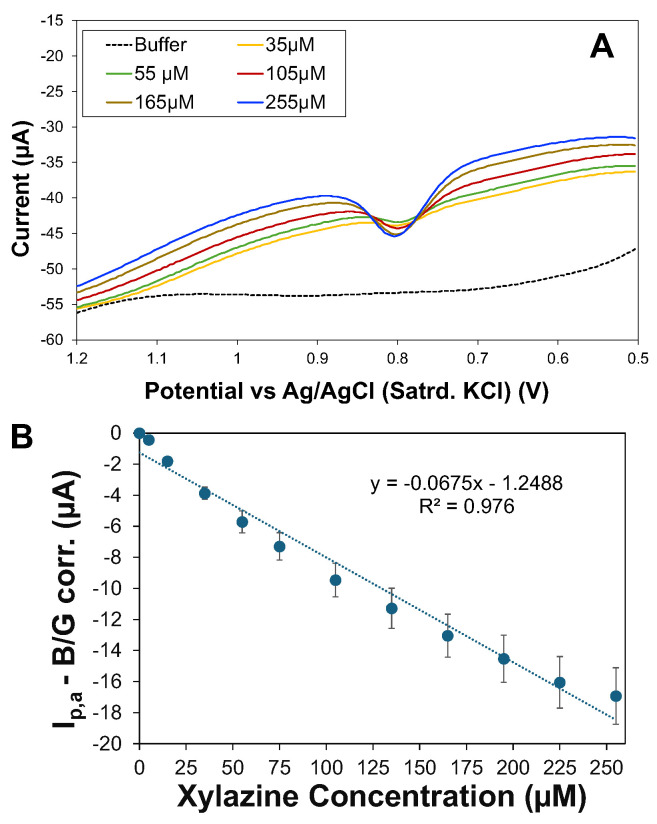
(**A**) Typical DPV scans collected with a modified GCE in increasing concentrations of XYL standard (50 mM) versus PBS background (*dashed*); (**B**) Representative standard calibration curve plotting background-corrected signal vs. XYL concentration. Note: For clarity, not all DPV scans at every XYL concentration are displayed in (**A**). Additional XYL calibration curve results are in the Supplemental Data.

Using DPV, XYL standard calibration curves were able to be generated with the fully modified electrodes, an example of which is shown in Figure 5. The performance of the sensor includes an average sensitivity of 67.5 µA/mM that, when normalized to the geometric area of the electrode, is calculated at 950 µA/mM∙cm^2^. The sensitivity projects across a linear range from about 15 to 255 µm and a reliable limit of detection (LOD) at under 5 ppm XYL. After calibration, the sensor response time to deliver a quantitative measurement of XYL is equivalent to a DPV scan across a limited potential window (<2 min). The analytical performance of the sensor, while comparable to other literature reports [30,31,32,33], has the additional advantages of being simple, cost-effective, robustly fabricated with readily available materials, and featuring an inherent resistance to fouling. For comparison purposes, analogous DPV in similar XYL concentrations at a bare GCE versus the modified electrode are provided in the Supplemental Data (Figures S7 and S8). As expected, the XYL oxidation peak potential shifts significantly at a bare GCE and there is a simultaneous loss of signal/current as XYL concentration is increased.

### 3.4. Application of Xylazine Sensor to Real Samples

#### 3.4.1. Detection of Street “Tranq”

**Figure 6:**
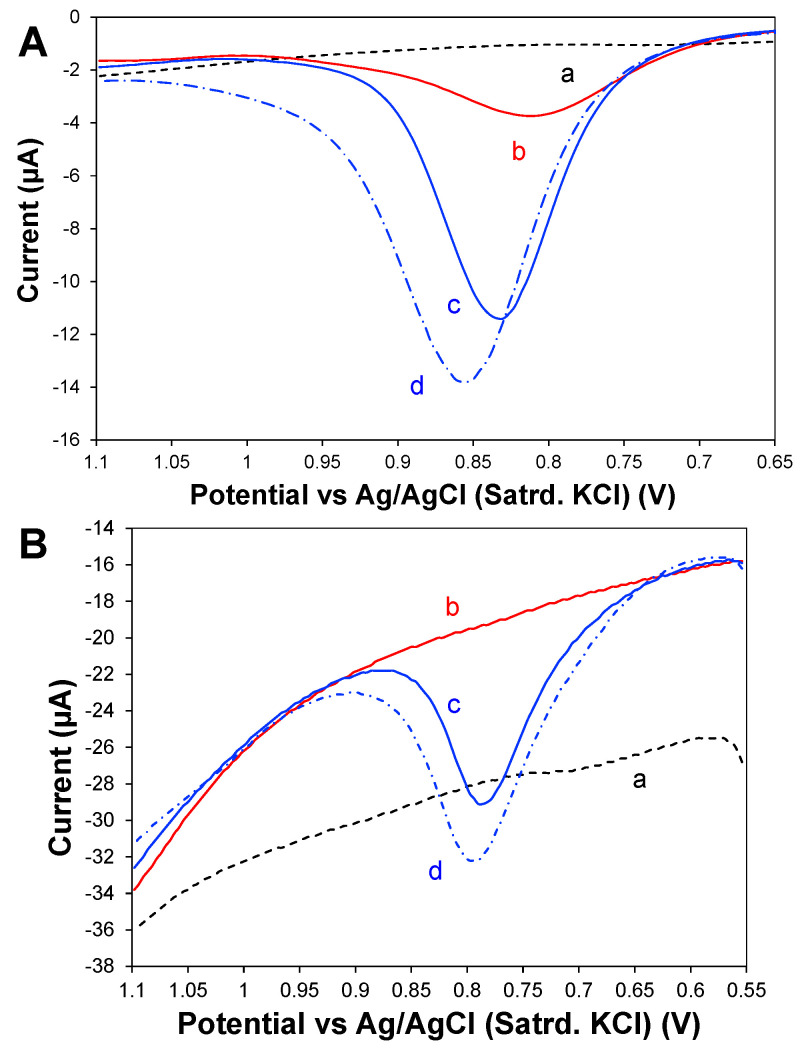
DPV scans of (**A**) bare and (**B**) fully modified GCEs in (a) 150 mM buffer (*dashed trace*), (b) 125 µM fentanyl, (c) a mixture of 125 µM fentanyl with 125 µM XYL and (d) that same mixture spiked with an additional 125 µM XYL spike or 250 μM XYL total concentration (*dashed-dot trace*).

One of the major objectives of this project was to create an electrochemical sensor that could be used by first responders or forensic investigators to quickly identify the likely presence of the dangerous street drug known as “Tranq”, a lethal mixture of fentanyl or one of its derivatives with XYL. The challenge of an electrochemical sensor is that fentanyl and numerous synthetic derivatives of fentanyl (e.g., methoxy-acetylfentanyl, isobutyrfentanyl, etc.) are oxidized at similar potentials to that of XYL [7,8]. Research has showed that the tertiary amine at position N1 (see Figure 1) of fentanyl, a structural component common to many of its synthetic derivatives as well, undergoes oxidation at nearly the same potential as XYL [6,48]. As such, an effective “Tranq” sensor requires signal differentiation between these synthetic opioids and XYL. Because of the overlapping oxidation potentials, however, a modified electrode must effectively exclude the fentanyl-associated redox chemistry. Figure 6 illustrates the performance of the modified electrode versus a bare GCE electrode in the presence of these compounds. At the bare GCE electrode, fentanyl is clearly oxidized at a similar potential to XYL while the mixture of fentanyl and XYL at bare GCEs shows a large peak that is the combination of the oxidation current from both compounds, a signal that cannot be easily deconvoluted (Figure 6A). However, at the modified electrode developed in this study, there is no peak observed from fentanyl, suggesting that the DPV of the mixture represents an isolated signal only from XYL. Considering the potency of fentanyl and derivatives of fentanyl, they are likely to comprise a smaller percentage of street drugs that have been adulterated with XYL. As such, it is important that the sensor is able to detect XYL at different ratios versus the fentanyl. From these results (Figure 6B), when the mixture is spiked to be 2:1 for XYL:fentanyl, the signal increases in size at the modified electrode. Notably, at the bare electrode, the extra spike of XYL results in a more significant shift in the peak due to fouling (Figure 6A). In summary, the modified electrode sensor is effective at detecting XYL even in presence of fentanyl at different ratios.

**Figure 7:**
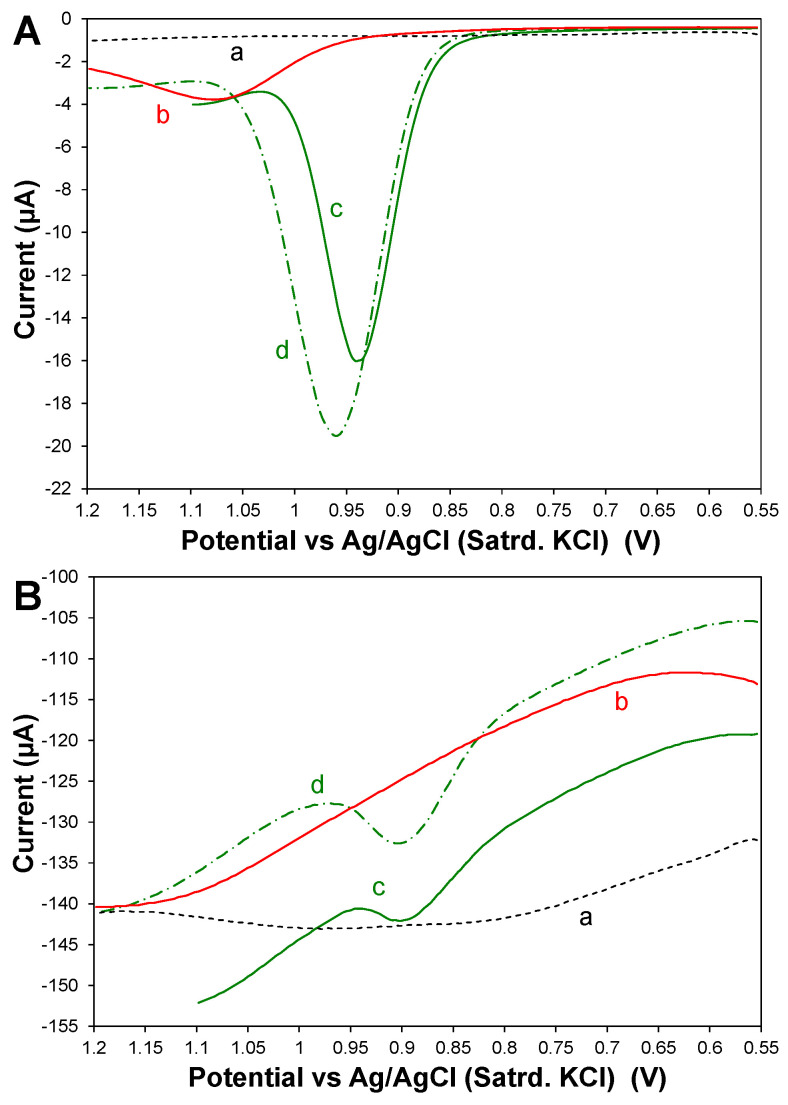
DPV scans of (**A**) bare and (**B**) fully modified GCEs in (a) 150 mM buffer (*dashed trace*), (b) 165 µM cocaine, (c) a mixture of 165 µM cocaine with 165 µM XYL and (d) that same mixture spiked with an additional 165 µM XYL spike or 330 μM XYL total concentration (*dashed-dot trace*).

Drug abuse can also involve other forms of “speedballs” or mixtures of drugs. Other non-opioid narcotics such as cocaine, for example, have very similar oxidation potentials to XYL and fentanyl at carbon electrodes [8]. The modified electrodes in this study had an analogous performance at mixtures of XYL and cocaine in different ratios (Figure 7). Again, as cocaine is likely to be the more minor component of the actual drug, the sensors were able to indicate the presence of XYL at different XYL:cocaine ratios, suggesting that the sensors remain quantitatively viable regardless of the mixture. Additionally, the modified electrode design effectively discriminates against the cocaine redox chemistry and can isolate the XYL signal.

Of note from the results is the often-observed electrocatalytic effect attributed to the incorporation of the CNTs within the modified electrode systems that typically shifts the oxidation of XYL to lower positive potentials versus the bare electrode (Figure 4, for example). The shift in oxidation potential for XYL, while overlapping with fentanyl (Figure 6), allows for delineation of a separate peak potential for cocaine oxidation (Figure 7) at ~1.125 V. As seen in other electrochemical studies, this oxidation potential is quite typical for a range of different non-fentanyl opioids. As such, the results suggest that it may be possible to employ both a modified and bare set of electrodes for a tandem measurement that would directly signal the presence of XYL (and an indirect likelihood of fentanyl or a fentanyl derivative) while also indicating the presence of a non-fentanyl opioid such as cocaine. As a preliminary step toward such a device, we tested bare and modified electrodes in mixtures of 2:1 cocaine-to-fentanyl and 2:1 fentanyl-to-cocaine samples with and without XYL (Supplemental Data, Figures S9 and S10). While this strategy requires more development, the preliminary results suggest that such dual measurements would yield more information about a sample, keeping in mind that the bare electrodes still quickly become fouled. In any event, the main take away message is the demonstrated ability to indirectly signal the presence of any number of the many fentanyl derivatives because of the detection of XYL adulterant.

#### 3.4.2. Detection of Xylazine in Beverages

In addition to abuse as a fentanyl adulterant in “Tranq”, XYL is also a common sexual assault drug that is doped into beverages [14,20,33]. For testing this application of the XYL sensors, a number of common drinks were selected for evaluation, including well-known brands of soda (i.e., cola), diet soda (i.e., diet cola), vodka, and tequila. A key issue when testing beverages is their potentially complex matrices and the evaluation of key interferent species that may obscure or conflict with the XYL oxidation peak used for quantification. A systematic approach was followed in that specific compounds common to a sample such as cola (e.g., caffeine, sucrose, glucose) or a diet cola (e.g., phenylalanine, aspartame, citric acid, acesulfame) were individually tested for electrochemical oxidation at a bare GCE that may interfere with the XYL signal. These results, presented in the Supplementary Data (Figure S11), revealed no major electroactivity from potential interferents in the +0.8 to +1.0 V potential window where XYL oxidation is observed. Similar preliminary testing with analogous findings was performed with vodka and tequila samples as well (Supplemental Data, Figure S12). Even with these preliminary results, however, we included quantitative analysis of simulated cola and diet cola doped with XYL in addition to applying the sensors to actual beverage samples. Details of sample preparation and beverage testing are supplied in the Experimental Details while the calibration curves generated in each matrix are provided in the Supplemental Data (Figures S13–S16). Table 1 summarizes the results of using our sensor to detect a spike of 125 µM XYL in different beverages compared to its established detection in PBS. As can be seen in the results, the sensors perform admirably in most of the beverages, where percent recovery was between 95 and 104%. The results suggest the most complicated matrix is the regular cola, which exhibited a significant loss of sensitivity. In some cases, when soda samples were first diluted (PBS), sensitivity and percent recovery increased. The exact reason for the complications in cola soda are unknown. If a specific interferent can be identified, the additional semi-permeable layers have been successfully employed to discriminate against individual compounds such as ascorbic acid [49]. Alternatively, soda cola samples may be approached with the electrodes via standard addition methodology, which has shown to improve detection of XYL in complex matrices [30,31].

## 4. Conclusions

With the recognition that fentanyl derivatization represents a significant complication to drug sensor development for first responders, medical personnel, police, and forensic investigators, the dangerous and pervasive adulteration of fentanyl-based street drugs with XYL, while lethal, provides an opportunity for more effective preliminary screening methods [3,50]. The widespread use of XYL as an adulterant in fentanyl and fentanyl derivatives provides an opportunity to design a sensor that circumvents the need to target so many structurally different but equally potent forms of fentanyl drugs. The oxidation of XYL additives represents a signal that can be used for fast identification of the compound and the indirect indication of co-existence of fentanyl or one of its many synthetic derivatives [6].This type of screening test would be a valuable tool to not only protect first responders but to also help inform their assessments and first treatments (e.g., administration of naloxone, an opioid antagonist, or tolazine to reverse effects of XYL). The sensor developed in this study is resistant to the notorious XYL fouling of electrodes during oxidation [30,32,33] and can detect XYL even in the presence of fentanyl and other opioids (e.g., cocaine). In addition to the fouling resistance of the presented sensor, it also benefits from simple construction of well-established and easily obtained materials while utilizing a relatively simple electrochemical method that can be readily miniaturized as a portable device [51].

## Figures and Tables

**Table 1:** Xylazine Sensor Performance in Various Beverages.

System/Matrix	Sensitivity (µA/mM) ^a^	n	Avg. Percent Recovery (%) ^a,c^
PBS/Standard	−67.5 _(±4.2)_	3	102.1 _(±6.4)_
Simulated Cola	−41.5 _(±4.6)_	8	95.7 _(±10.7)_
Simulated Diet Cola	−36.3 _(±3.1)_	4	97.0 _(±8.2)_
Cola	−7.7 _(±0.9)_	4	59.2 _(±7.6)_
Diet Cola	−15.4 _(±2.0)_	7	95.6 _(±12.4)_
Diluted Cola ^b^	−69.4 _(±4.4)_	4	84.5 _(±6.3)_
Diluted Vodka ^b^	−42.2 _(±5.0)_	4	103.5 _(±12.3)_
Diluted Tequila ^b^	−23.5 _(±3.4)_	4	76.50 _(±11.1)_

Notes: ^a^ Uncertainty ( ) represents relative standard error; ^b^ Samples were diluted (1:1) with PBS; ^c^ Based on a 125 μM target concentration of XYL spike.

## Data Availability

The data that support the findings of this study are available from the corresponding author upon reasonable request.

## Supplementary Materials

The following supporting information can be downloaded at: https://www.mdpi.com/article/10.3390/toxics12110791/s1, Scheme S1: proposed example of XYL oxidation; Figure S1: XYL DPV with DEHP; Figures S2 and S3: CV analysis of each modifying layer; Figure S4: DVP oxidative scans at different XYL concentrations; Figure S5: Scan rate analysis of XYL voltammetry; Table S1: CV scan rate analysis results; Figure S6: XYL DPV repeated scans with fouling effect; Figures S7 and S8: DPVs used for XYL calibration curves at modified and unmodified electrodes; Figures S9 and S10: DPV scans in different mixtures of cocaine, XYL, and fentanyl; Figures S11 and S12: interferent analysis; Figures S13–S16: DPV generated calibration curves for cola, diet cola, vodka, and tequila solutions.
