# Supplementary material for: Fouling-Resistant Voltammetric Xylazine Sensors for Detection of the Street Drug “Tranq”
Source: Toxics. 2024 Oct 30;12(11):791. doi: 10.3390/toxics12110791 (PMC11598047; doi:10.3390/toxics12110791)
Supplement: Supplementary file 1 [file toxics-12-00791-s001.zip › toxics-3269621-supplementary.pdf]

Supplemental Data: **Fouling-Resistant Voltammetric Xylazine Sensors for Detection of the Street Drug "Tranq"**

Joyce E. Stern, Ann H. Wemple, Arielle Vinnikov, Charlie W. Sheppard, and Michael C. Leopold*

**Department of Chemistry, Gottwald Center for the Sciences, University of Richmond, Richmond, Virginia 23173, United States (Corresponding Author)*

Table of Contents for Supplemental Data:

- **Scheme S1**: One proposed mechanism of XYL oxidation
- DPV oxidative scans of XYL, DEHP, and a mixture showing competition for β-CD cavities (selectivity) (**Figure S1**).
- CV analysis of each layer/components effect on voltammetry signal including layer-by-layer (**Figure S2**) and voltammogram overlays (**Figure S3**).
- DPV oxidative scans of 0, 1, and 5 mM XYL solutions showing the concentration dependence and independence of oxidation peaks at ~ +0.9 and ~ 0.0 V, respectively (**Figure S4**).
- Small potential window scan rate analysis of voltametric peaks including XYL oxidation and reduction of its product (**Figures S5-A/B** and **Table S1**).
- Repeated DPV oxidative scans of bare and modified GCE in 255 μM XYL showing peak current, peak potential, and fouling effects to XYL exposure (**Figure S6**).
- DPV oxidative scans of a modified GCE in increasing standard XYL concentrations for generating a calibration curve (**Figure S7**).
- DPV oxidative scans of a bare GCE in increasing standard XYL concentrations for generating a calibration curve (**Figure S8**).
- DPV scans of tandem result using both a bare and modified electrode system in different mixtures of XYL, cocaine, and fentanyl (**Figures S9-S10**).
- DPV oxidative scans of XYL and potential interferent species found in solutions of cola and diet cola (**Figure S11**).
- DPV oxidative scans of XYL and potential interferent species found in solutions of vodka and tequila (**Figure S12**).
- DPV generated calibration curves for beverage solutions including simulated soda and diet soda (**Figure S13**), real samples of cola and diet cola (**Figure S14**), diluted cola samples (**Figure S15**), as well as tequila and vodka solutions (**Figure S16**).

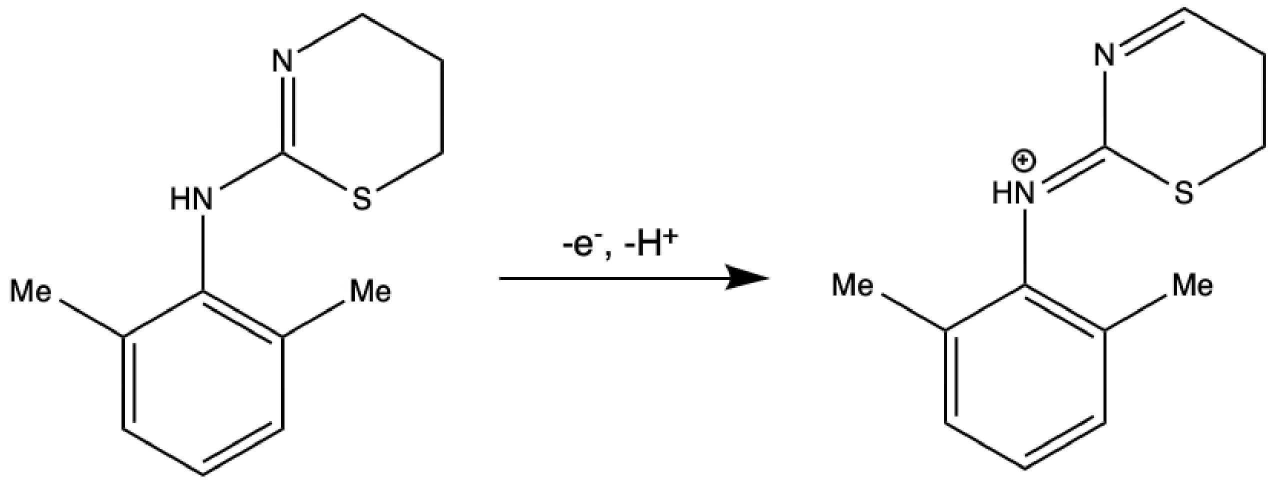

Scheme S1. One of the more commonly proposed mechanisms for the oxidation of XYL at an electrode (References Note: As mentioned in the text, there is no consensus on the exact electrochemical mechanism and/or redox chemistry of XYL, with some reports indicating different multi-step processes, for example.

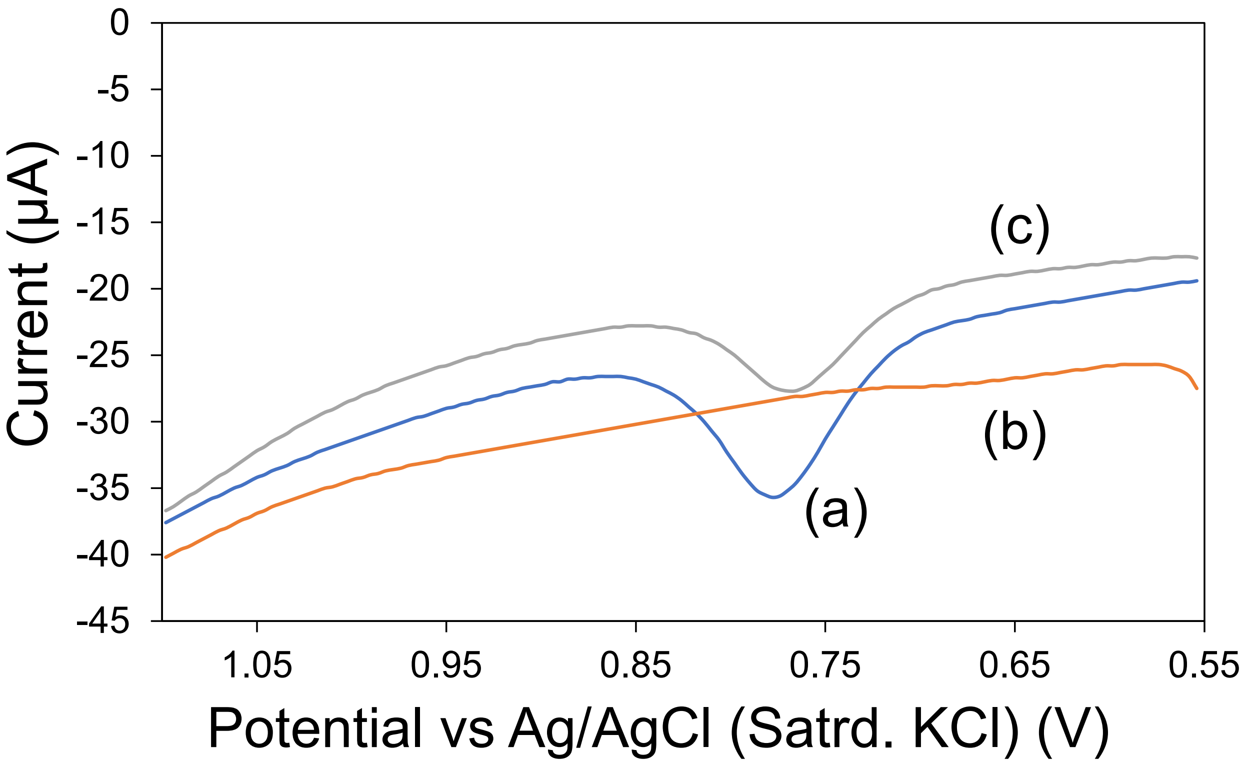

Figure S1. DPV oxidative scans of modified GCEs in a solution of (a) 125 µM XYL, (b) 250 µM Di(2-Ethylhexyl)Phthalate (DEHP), and (c) a mixture of 125 µM XYL and 250 µM DEHP showing that peak area is diminished as the DEHP blocks the β-CD binding sites stronger than XYL. Note: All solutions are in 150 mM PBS (pH = 7).

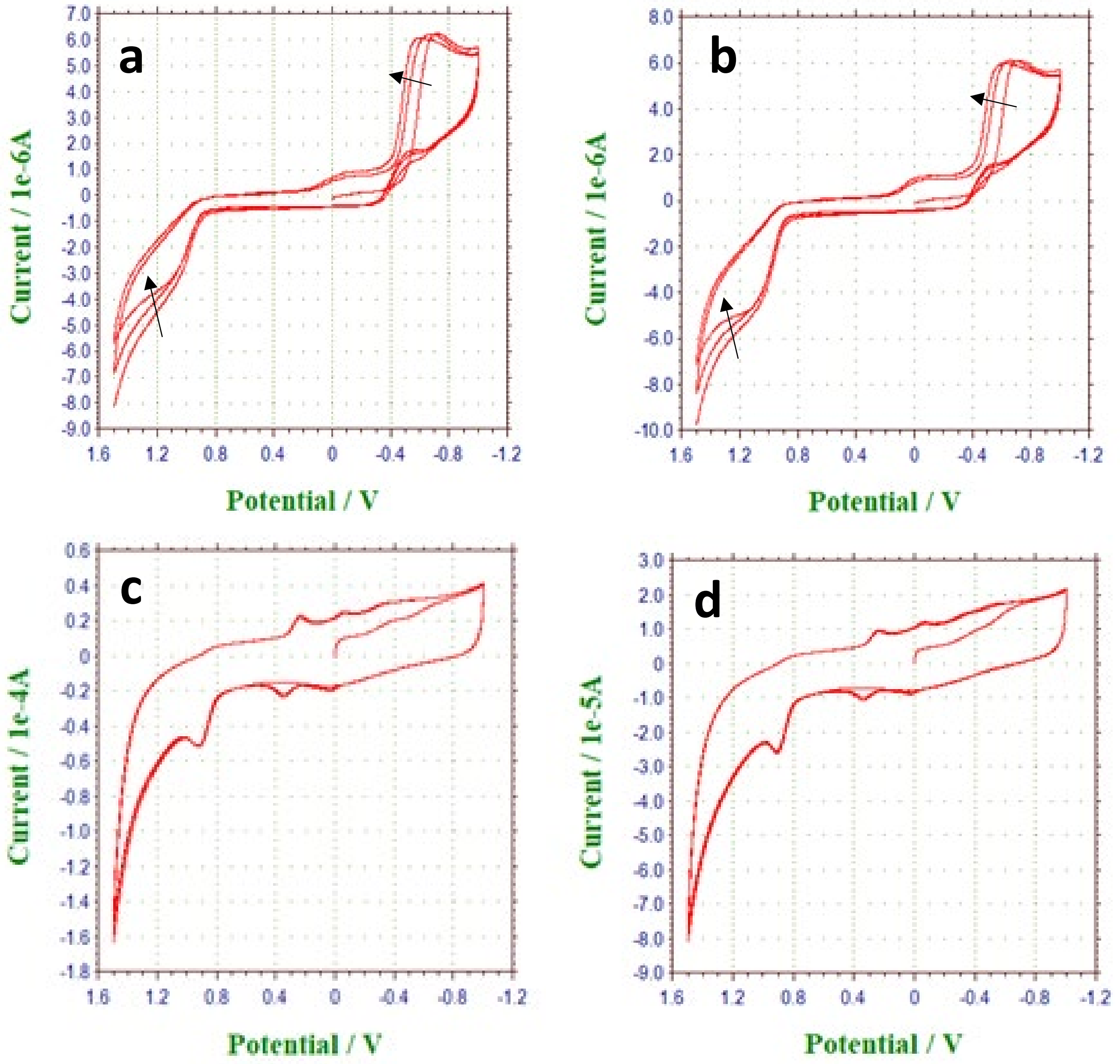

Figure S2. Cyclic voltammetry (3 cycles; 6 segments) of various systems (modified electrodes) in 1 mM XYL (150 mM PBS) including **(a)** GCE/PU; **(b)** GCE/β-CD/PU; **(c)** GCE/COOH-MWCNT/PU and; **(d)** GCE/COOH-MWCNT + β-CD/PU (full, optimized modified electrode system). Notes: In the presence of MWCNTs, XYL oxidation potential shifts to more negative potentials (scan rate is 50 mV/sec).

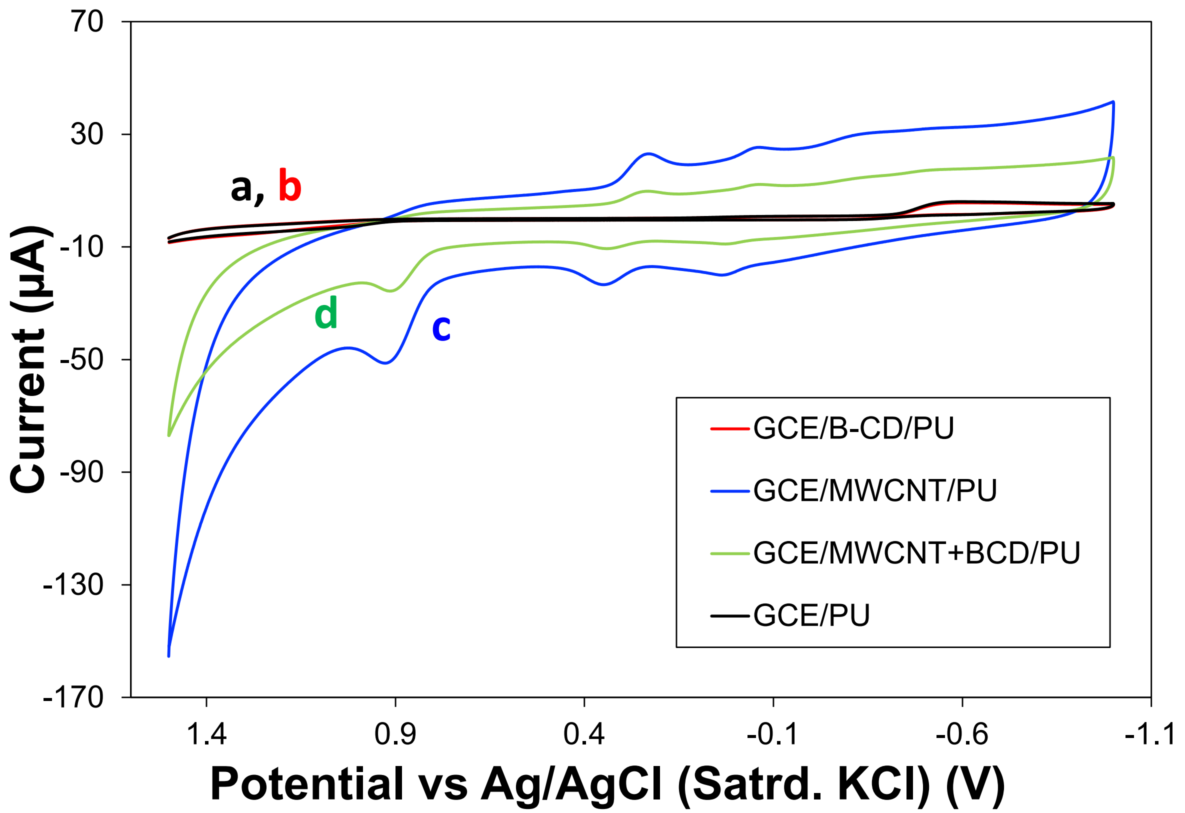

Figure S3. Overlay of cyclic voltammograms of various systems from Fig. S2 in 1 mM XYL (150 mM PBS) including **(a)** GCE/PU; **(b)** GCE/β-CD/PU; **(c)** GCE/COOH-MWCNT/PU and; **(d)** GCE/COOH-MWCNT + β-CD/PU (full, optimized modified electrode system). Note: scan rate is 50 mV/sec.

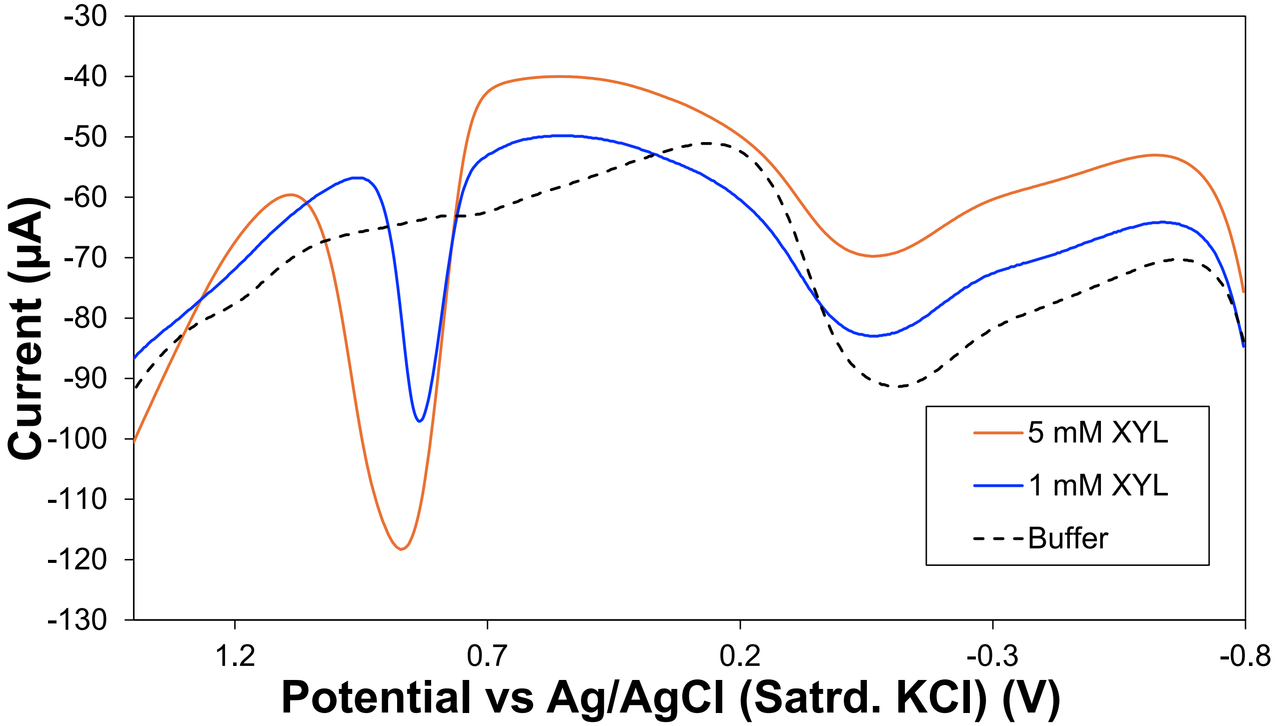

Figure S4. DPV oxidative scans of a fully modified electrode (GCE/COOH-MWCNT + β-CD/PU) in PBS (0 mM XYL), 1 mM XYL, and 5 mM XYL showing that the oxidation peak at ~+0.9 V is sensitive to concentration while the oxidation peak ~+0.0 V is present without XYL present and is not sensitive to changes in XYL concentration.

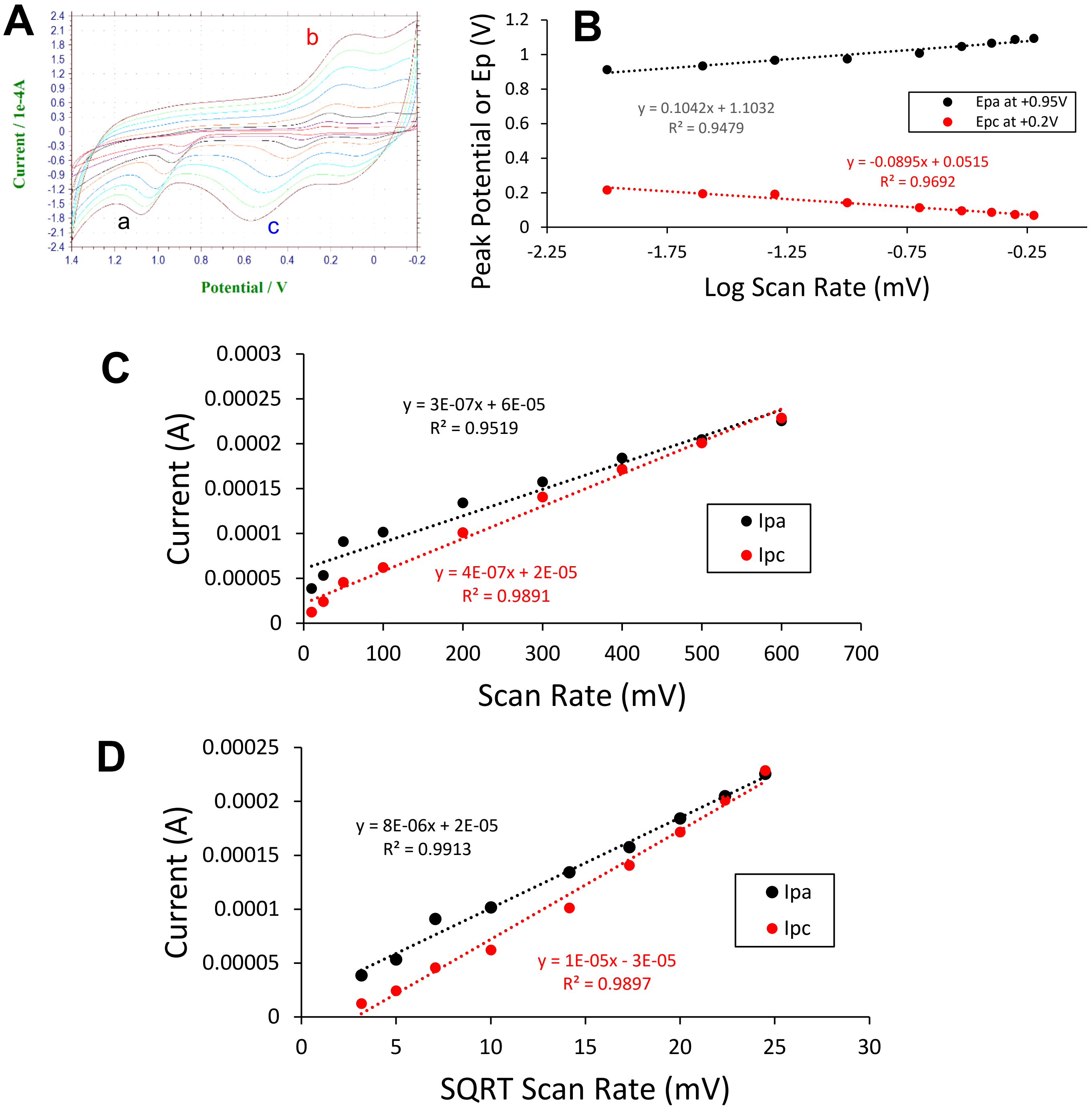

Figure S5-A. (A) scan rate CVs (Fig. 3) identifying **peaks a and b** analyzed for scan rate dependence (a = XYL oxidation; b = reduction of XYL oxidation product; c = MWCNT oxidation); Example plots for **(B)** E_p vs Log v; **(C)** I_p vs v (adsorbed) and; **(D)** I_p vs √v (diffusional).

A

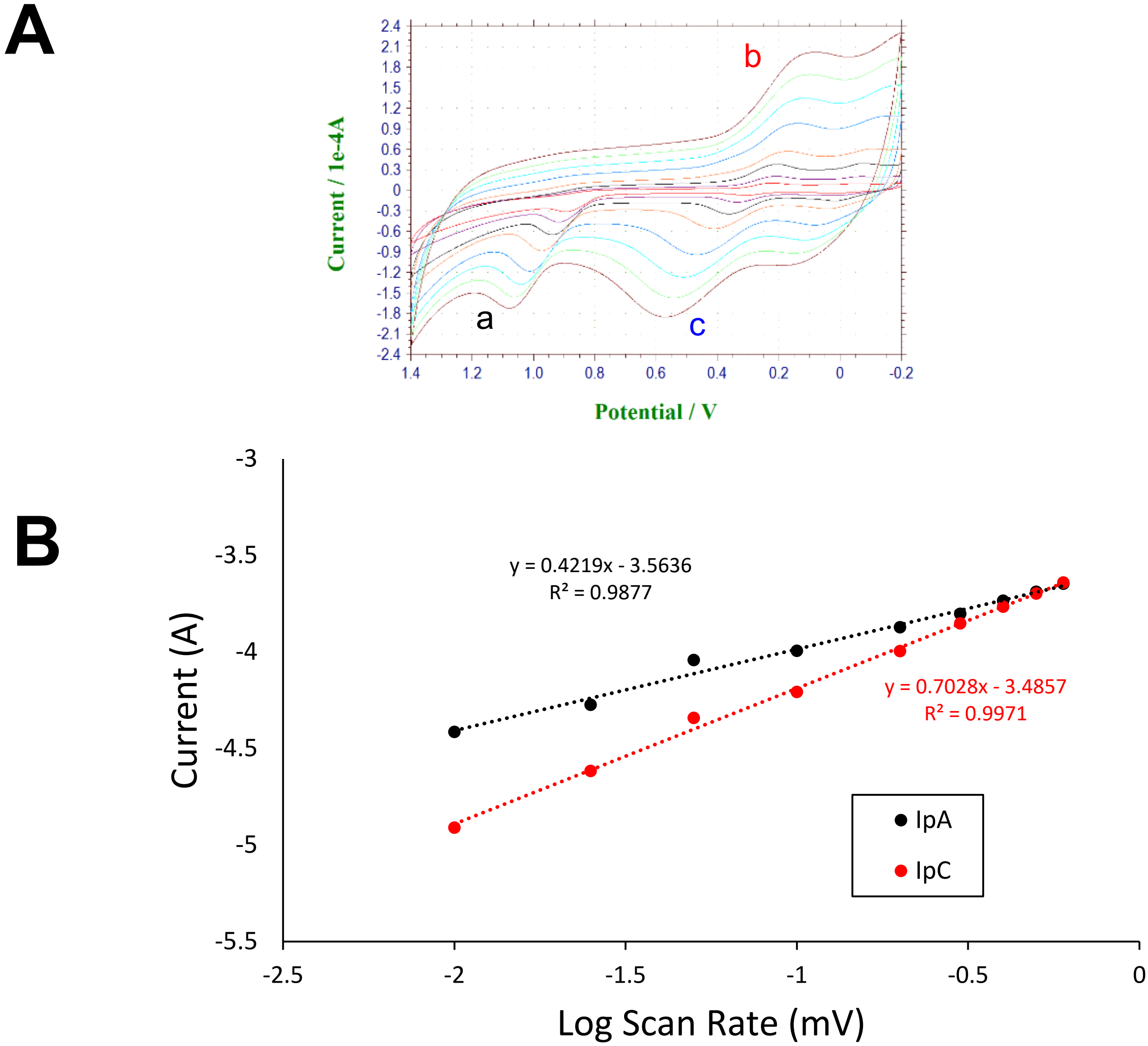

Figure S5-B. (A) scan rate CVs (Fig. 3) identifying **peaks a and b** analyzed for scan rate dependence (a = XYL oxidation; b = reduction of XYL oxidation product; c = MWCNT oxidation); **(B)** Example log(v)-log (i_p) plot. Note: Peak c (at ~+0.3 originally) was analyzed in the same manner with results summarized in Table S1.

Table S1: Avg. values for CV scan rate (v) dependence of 2.5 mM XYL/PBS

Parameter	(a) Anodic peak at ~ $^{+}$0.95 V	(b) Cathodic peak at ~ $^{+}$0.2 V	(c) Anodic peak at ~ $^{+}$0.3 V
i_p vs. ν (R^2)	0.9220 (±0.0307)	0.9911 (±0.0014)	0.9806 (±0.0090)
i_p vs. $\sqrt{\nu}$ (R^2)	0.9815 (±0.0136)	0.9882 (±0.0011)	0.9912 (±0.0005)
log i_p vs. log ν (slope)	0.4210 (±0.0156)	0.7094 (±0.0316)	0.8007 (±0.0303)
log i_p vs. log ν (R^2)	0.9794 (±0.0128)	0.9916 (±0.0033)	0.9978 (±0.0013)
Suggested Behavior (adsorbed vs. diffusional)	Diffusional	Adsorbed and Diffusional (mixed)	Adsorbed and Diffusional (mixed)

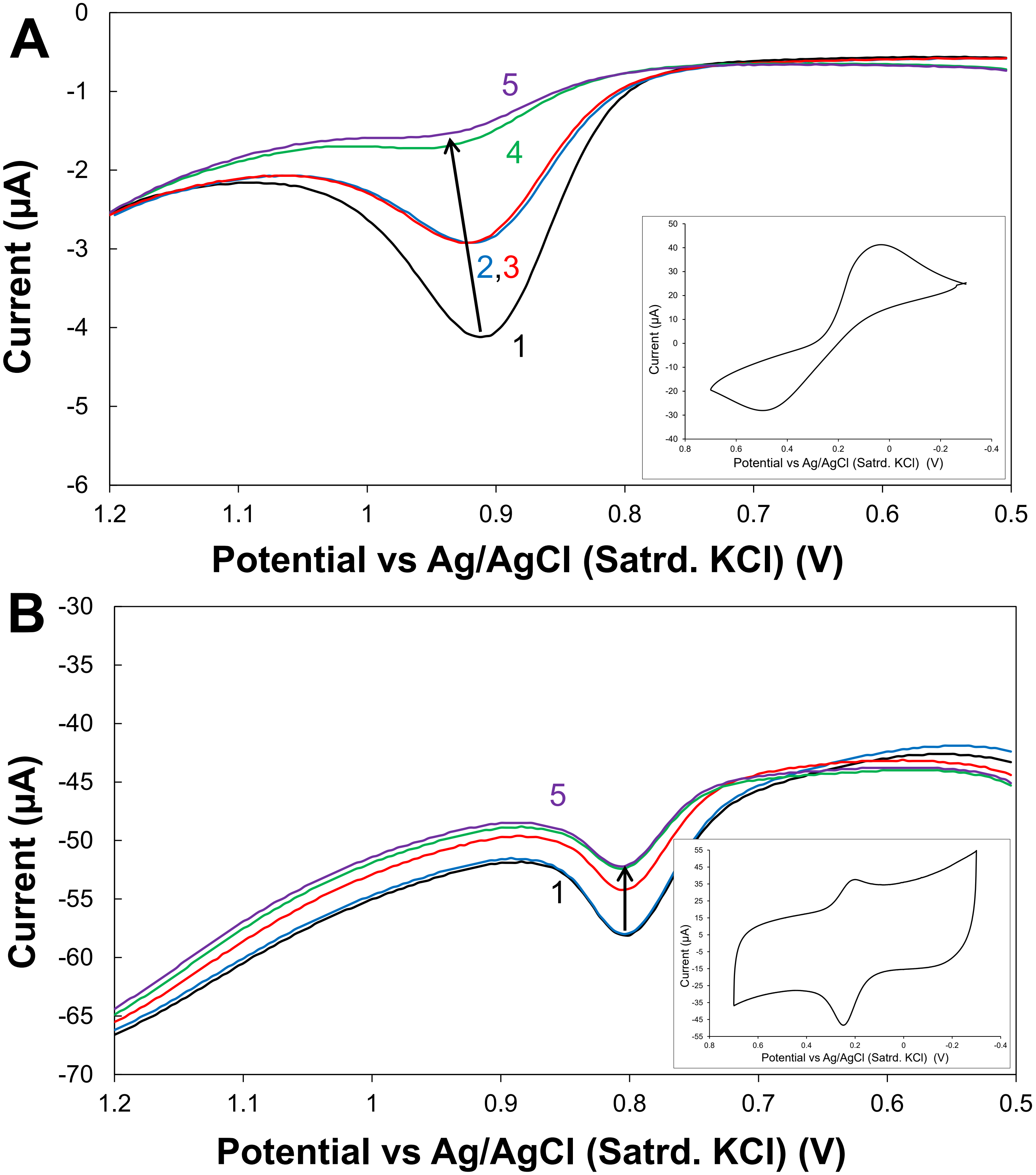

Figure S6. Consecutive DPV scans (5) in 255 µM XYL in PBS (150 mM; pH = 7) at a **(A)** bare and **(B)** modified GCE showing relative decreases in peak current and peak potential shifts with successive scans; the relative fouling effects are captured by CV of 5 mM potassium ferricyanide (100 mV/sec) after the 5 DPV oxidative scans (**insets**). Note: Peak definition and an order of magnitude higher current is maintained at the DPV for the modified electrode with essentially no peak potential shift.

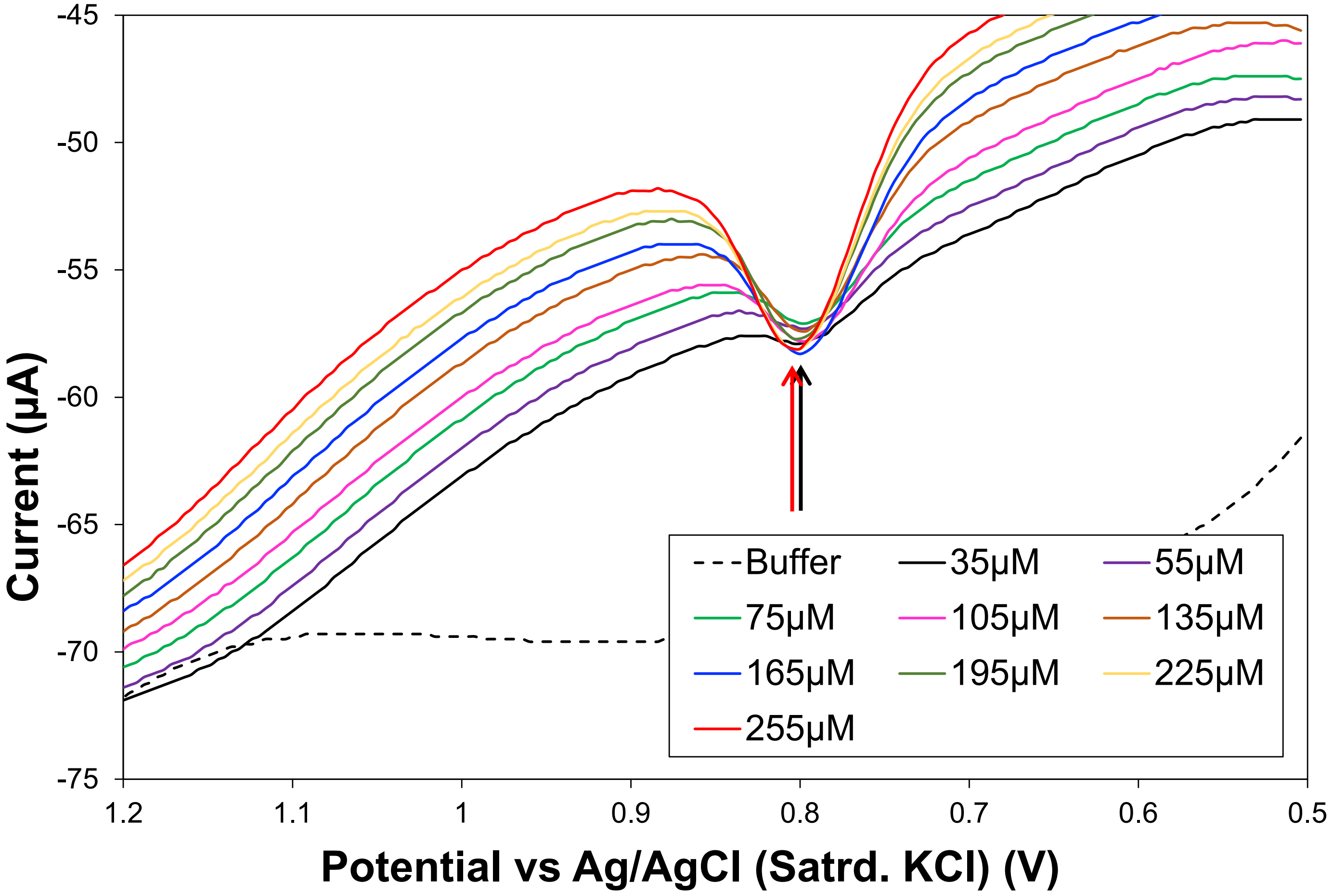

Figure S7. All (DPV oxidation scans (all) of a modified GCE exposed to increasing concentrations of XYL to create a standard calibration curve. Note: Only a slight shift in the $E_{p,a}$ is observed (black and red arrows).

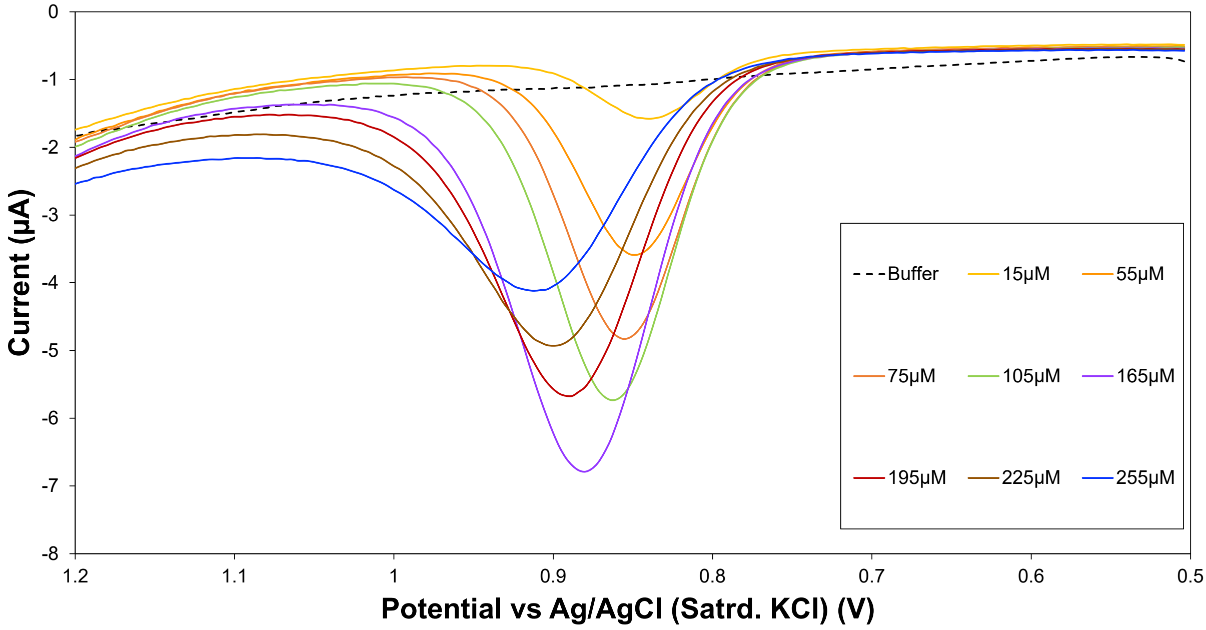

Figure S8. DPV oxidation scans (all) of a bare GCE exposed to increasing concentrations of standard XYL. Note: As standard XYL increases, there is a nearly immediate and consistent shift in peak potential while at a certain concentration (165 µM), the linear relationship between concentration and signal ceases as peak current decreases and peak shape broaden from fouling.

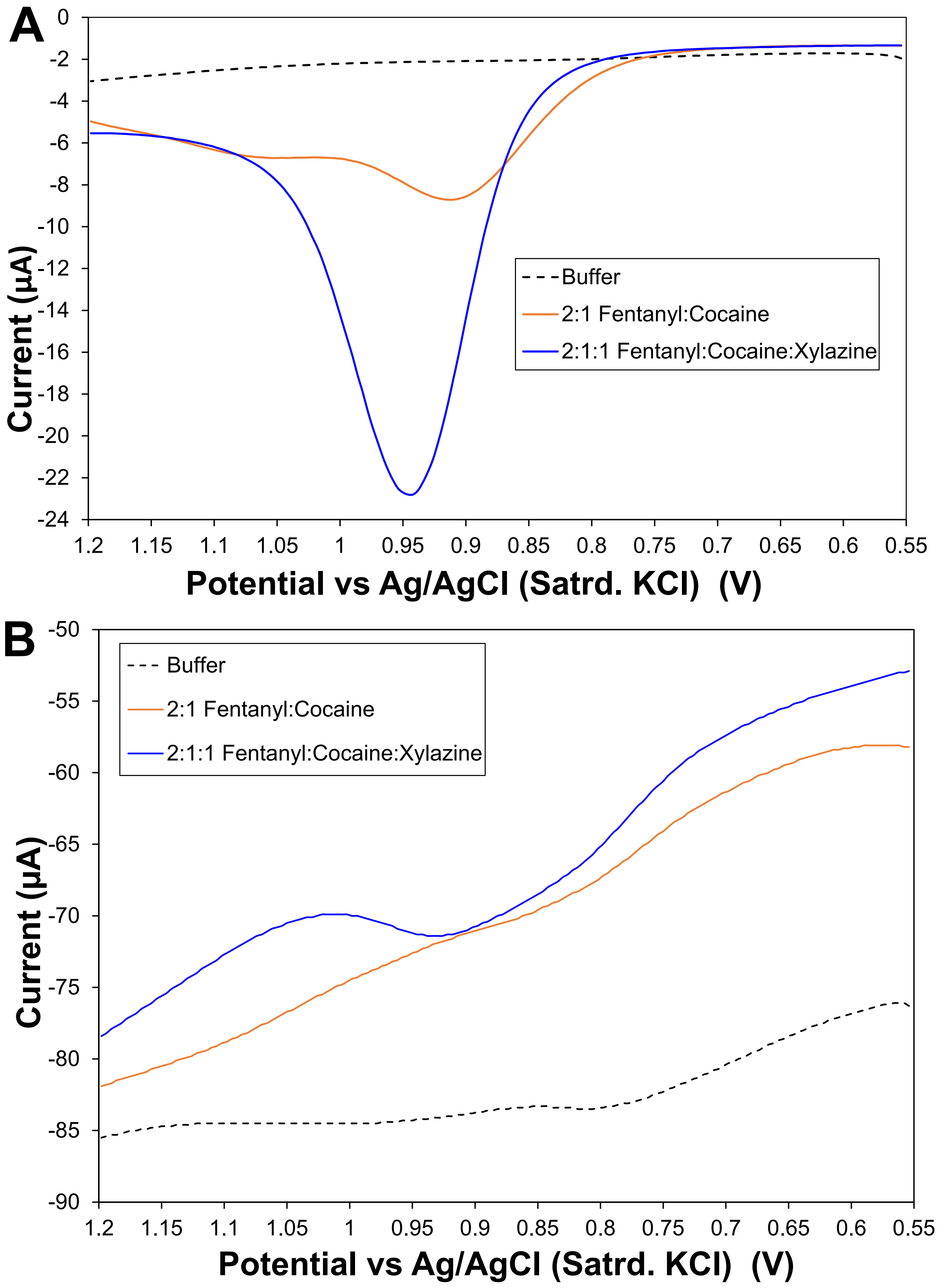

Figure S9. DPV scans of **(A)** bare GCE and **(B)** modified GCE in **(a)** 150 mM buffer, **(b)** a mixture of 2:1 fentanyl and cocaine (300 µM : 165 µM), and **(c)** a mixture of 2:1:1 fentanyl to cocaine to xylazine (300 µM : 165 µM : 165 µM).

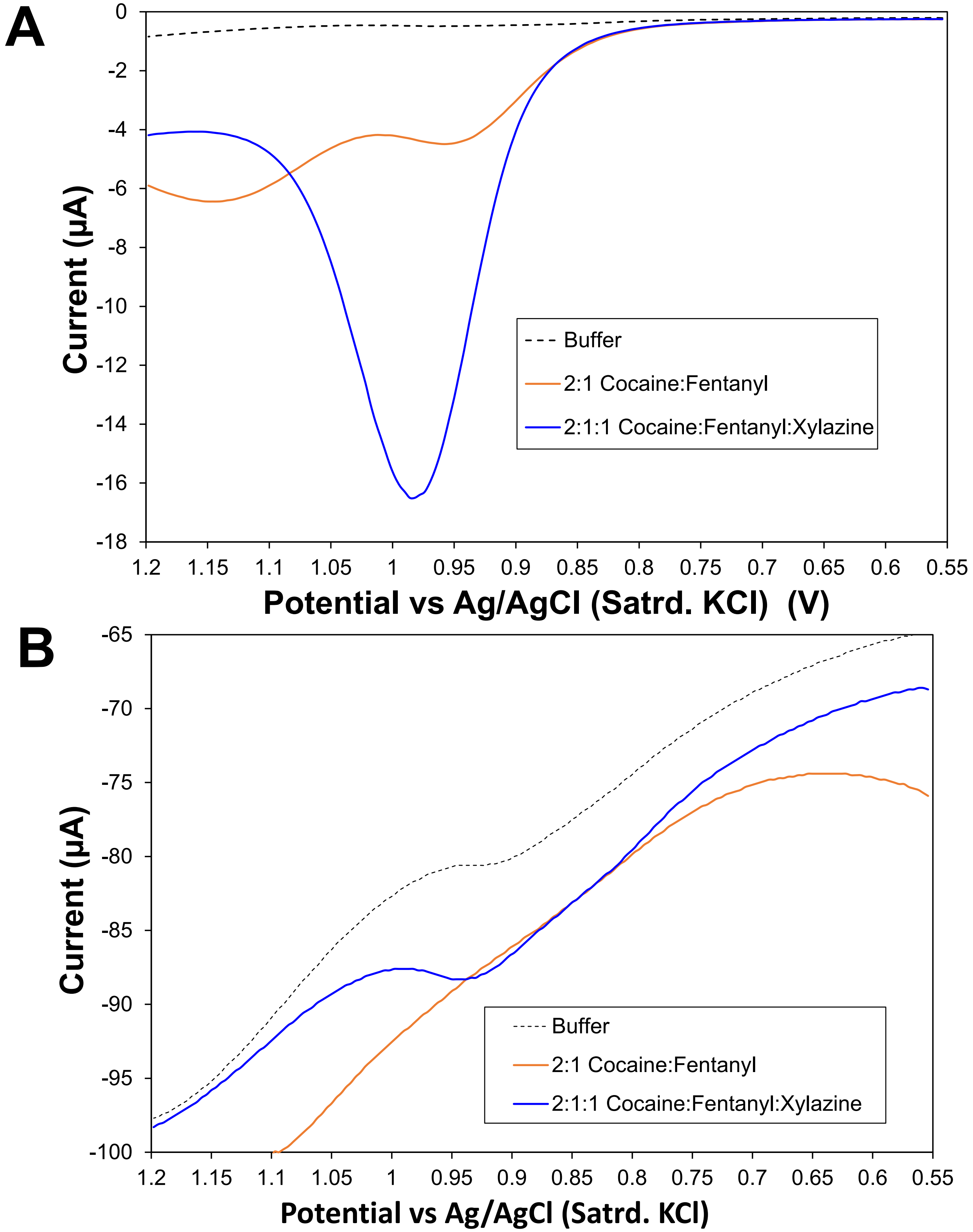

Figure S10. DPV scans of **(A)** bare GCE and **(B)** modified GCE in **(a)** 150 mM buffer, **(b)** a mixture of 2:1 cocaine and fentanyl (330 µM : 150 µM) and **(c)** a mixture of 2:1:1 cocaine to fentanyl to xylazine (330 µM : 150 µM : 150 µM).

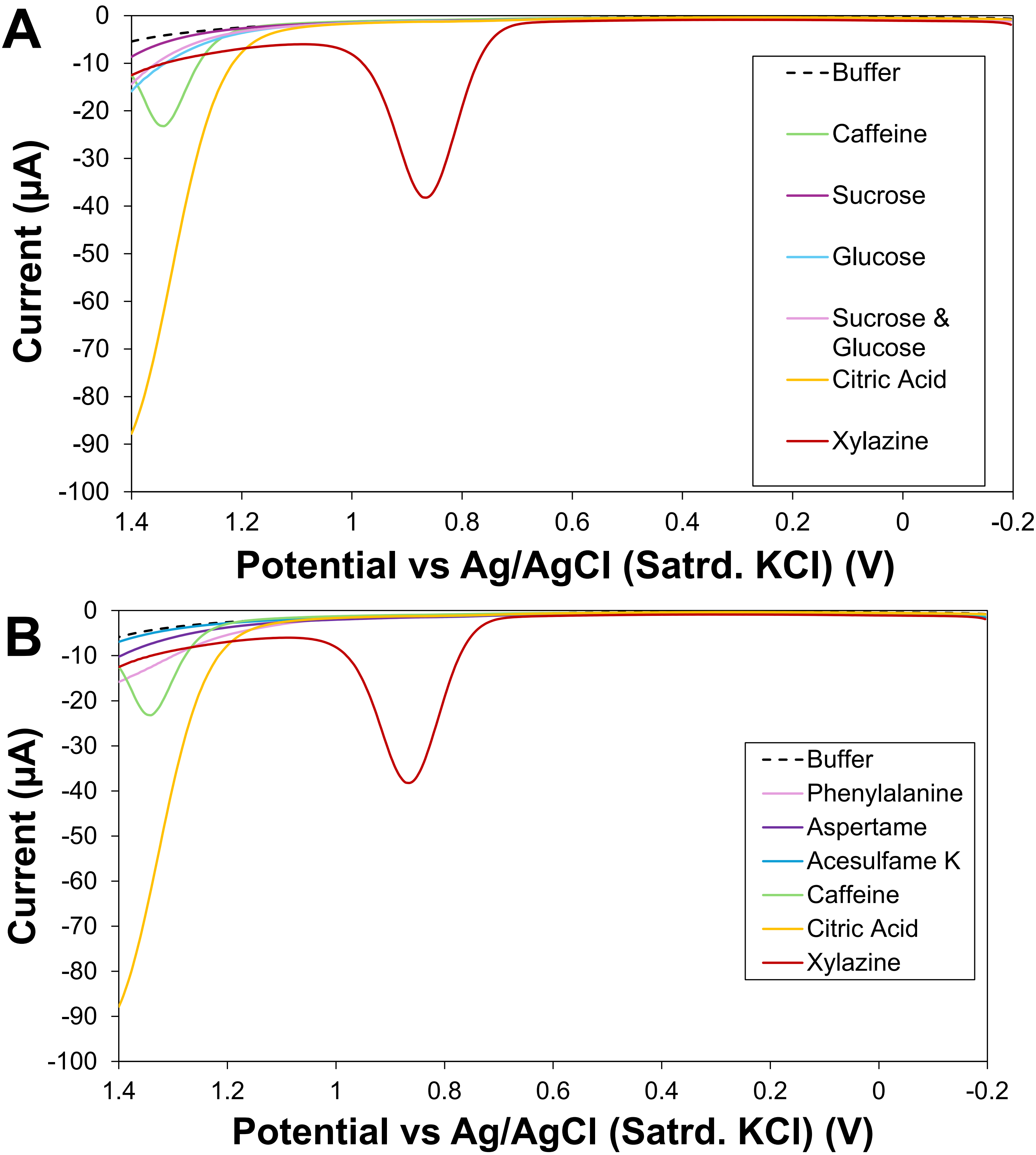

Figure S11. DPV oxidation scans of XYL (1 mM) and typical approximate concentrations of common components/interferents in **(A) regular soda:** caffeine (0.50 mM), sucrose (165 mM), glucose (313 mM), mixture of sucrose/glucose (165 and 313 mM), and citric acid (10 mM) and; **(B) diet soda:** caffeine (0.50 mM), citric acid (10 mM); phenylalanine (1.7 mM), aspartame (2.4 mM), and Acesulfame K (0.21 mM) at the modified electrode system.

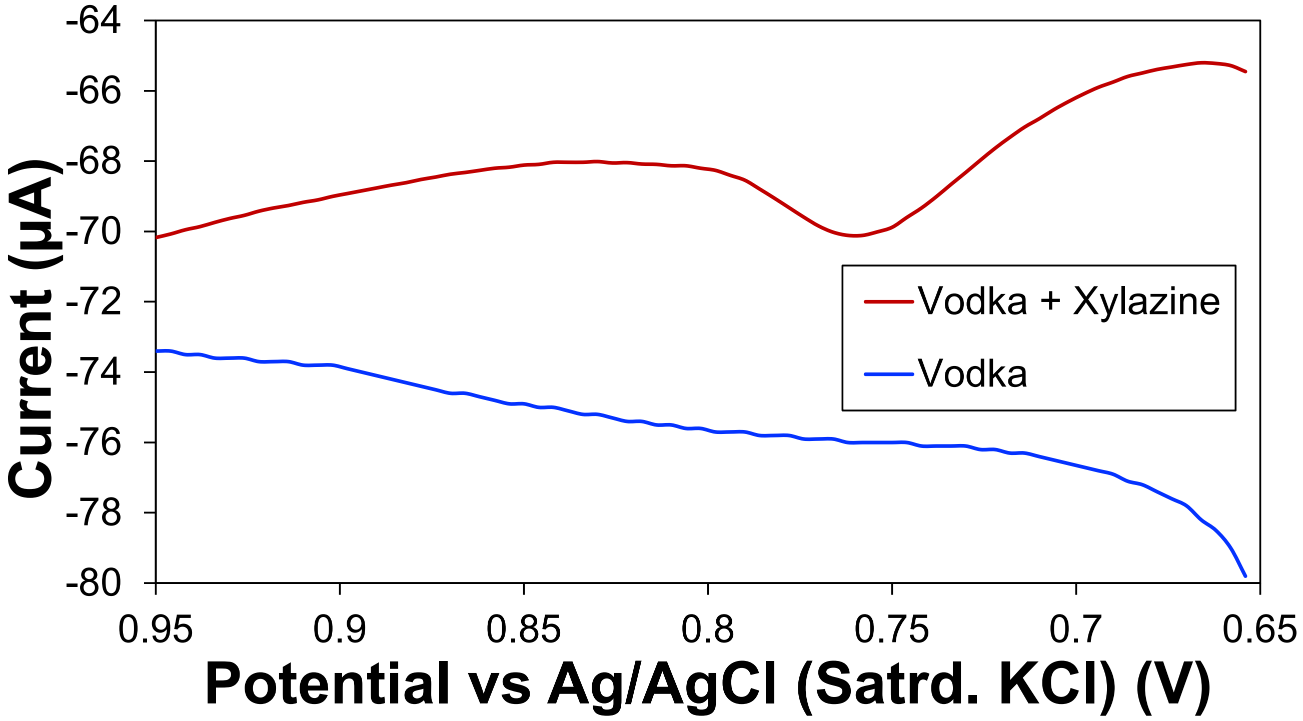

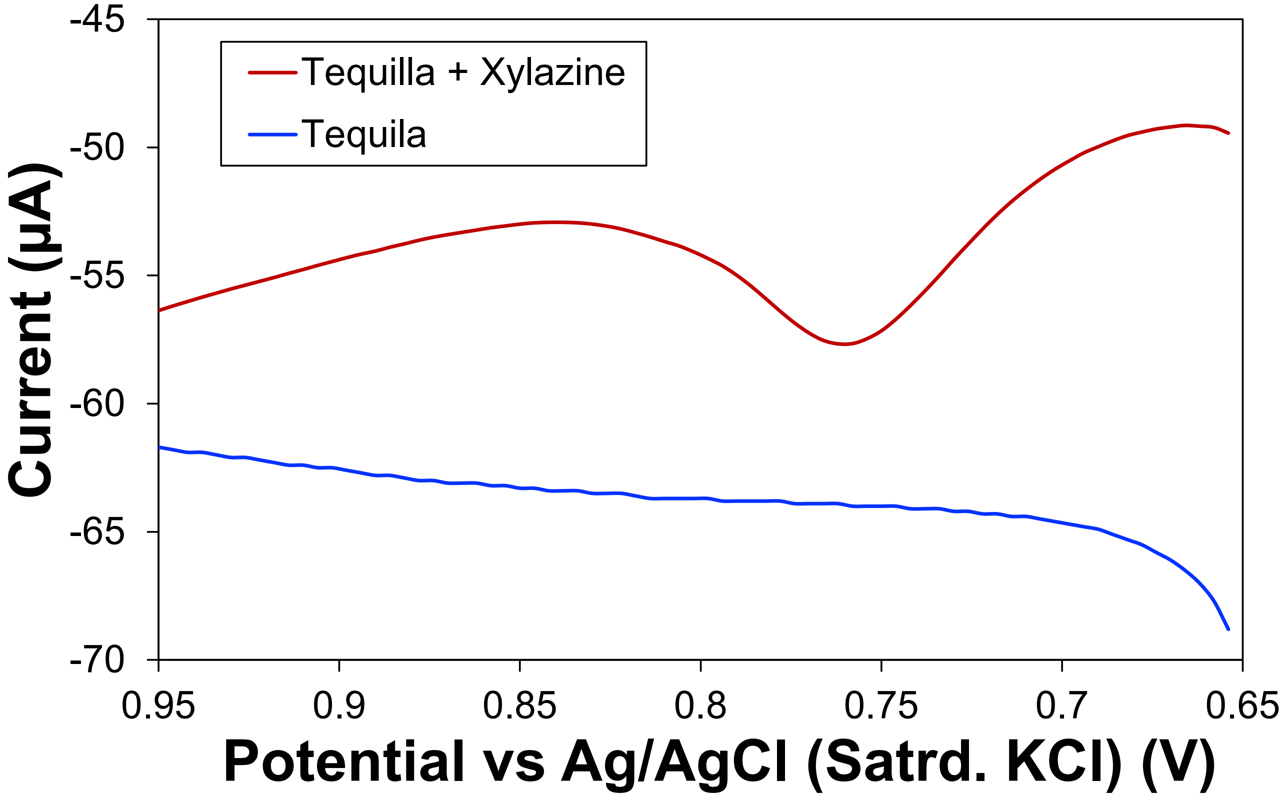

Figure S12. DPV oxidation scans of with and without XYL (125 µM) at the modified electrode system immersed in diluted (A) vodka and (B) tequila samples (1:1 with 150 mM PBS) with results suggesting no interferent-related voltammetric peaks from the beverage matrices in either case.

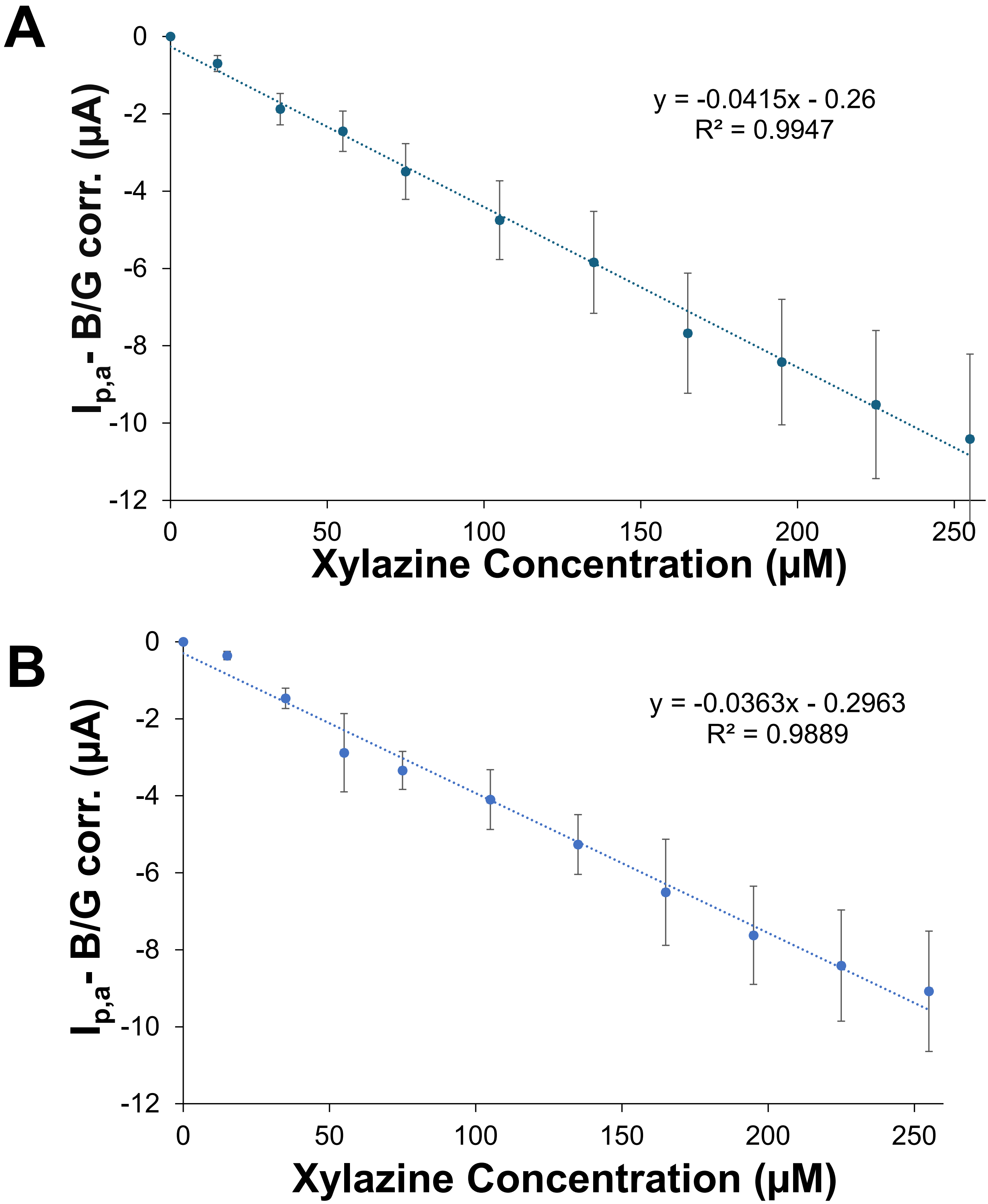

Figure S13. DPV generated XYL standard calibration curves for **(A)** simulated soda solution and **(B)** simulated diet soda solution.

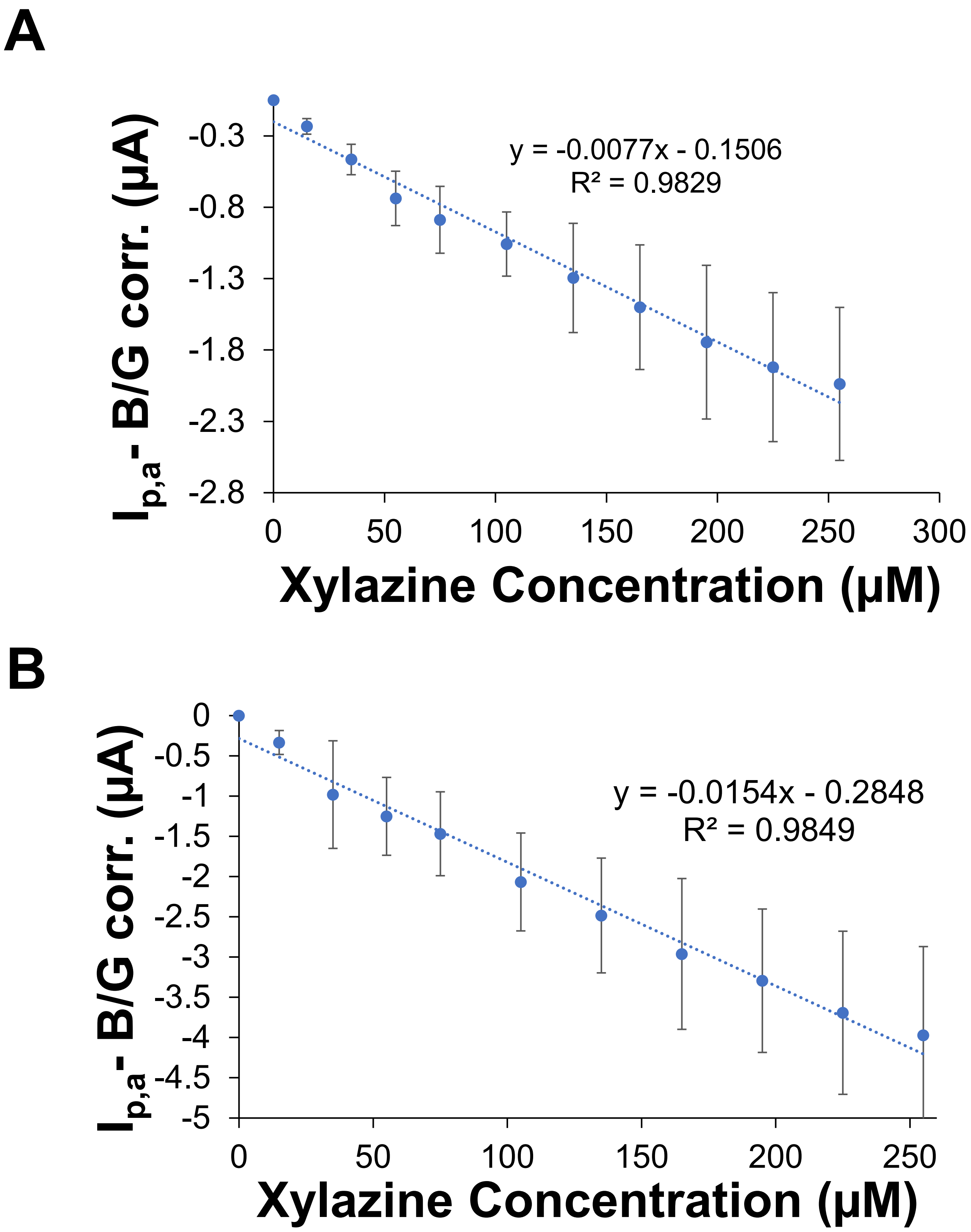

Figure S14. DPV generated XYL standard calibration curves for (A) cola and (B) diet cola solution (undiluted).

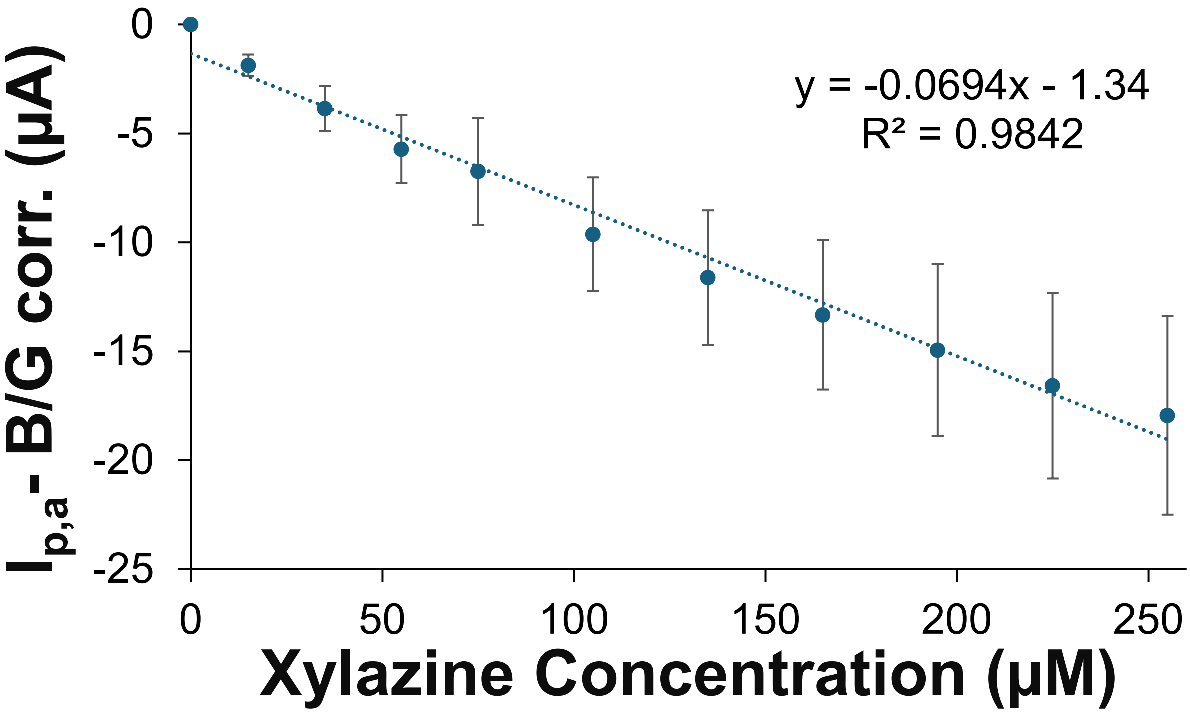

Figure S15. DPV generated XYL standard calibration curves for diluted (1:1, 150 mM PBS) cola.

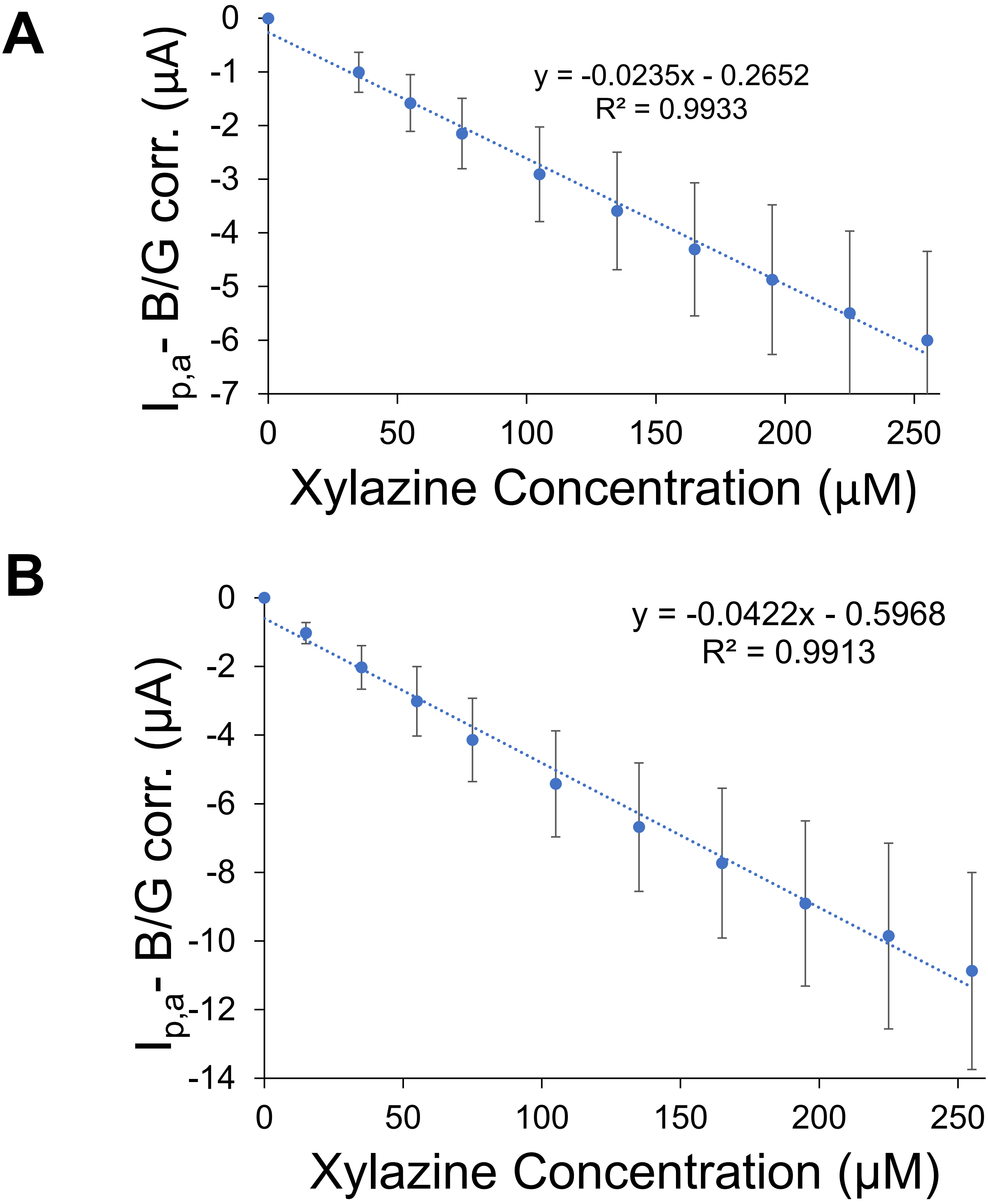

Figure S16. DPV generated XYL standard calibration curves for (A) tequila and (B) vodka solutions. NOTE: Beverages were diluted with 150 mM PBS in a 1:1 ratio .
